# A study of the interaction space of two lactate dehydrogenase isoforms (LDHA and LDHB) and some of their inhibitors using proteochemometrics modeling

**DOI:** 10.1186/s13065-023-00991-6

**Published:** 2023-07-06

**Authors:** Sedigheh Damavandi, Fereshteh Shiri, Abbasali Emamjomeh, Somayeh Pirhadi, Hamid Beyzaei

**Affiliations:** 1 Department of Bioinformatics, Laboratory of Computational Biotechnology and Bioinformatics (CBB Lab), University of Zabol, Zabol, Iran; 2 Department of Chemistry, Faculty of Science, University of Zabol, Zabol, Iran; 3 Department of Plant Breeding and Biotechnology (PBB), Faculty of Agriculture, University of Zabol, Zabol, Iran; 4 Medicinal and Natural Products Chemistry Research Center, Shiraz University of Medical Sciences, Shiraz, Iran

**Keywords:** Proteochemometrics, Machine learning algorithm, Isoenzyme, *Camb* package, Morgan fingerprints

## Abstract

Lactate dehydrogenase (LDH) is a tetramer enzyme that converts pyruvate to lactate reversibly. This enzyme becomes important because it is associated with diseases such as cancers, heart disease, liver problems, and most importantly, corona disease. As a system-based method, proteochemometrics does not require knowledge of the protein's three-dimensional structure, but rather depends on the amino acid sequence and protein descriptors. Here, we applied this methodology to model a set of LDHA and LDHB isoenzyme inhibitors. To implement the proteochemetrics method, the camb package in the R Studio Server programming environment was used. The activity of 312 compounds of LDHA and LDHB isoenzyme inhibitors from the valid Binding DB database was retrieved. The proteochemometrics method was applied to three machine learning algorithms gradient amplification model, random forest, and support vector machine as regression methods to find the best model. Through the combination of different models into an ensemble (greedy and stacking optimization), we explored the possibility of improving the performance of models. For the RF best ensemble model of inhibitors of LDHA and LDHB isoenzymes, and were 0.66 and 0.62, respectively. LDH inhibitory activation is influenced by Morgan fingerprints and topological structure descriptors.

**Supplementary Information:**

The online version contains supplementary material available at 10.1186/s13065-023-00991-6.

## Introduction

Cancer is one of the leading causes of death worldwide. Mutations in genes lead to the development of cancer when these mutations affect how cells function [1]. Tumor cells depend on glycolysis for ATP synthesis, even when oxygen is present to support oxidative phosphorylation, a process referred to as aerobic glycolysis or the Warburg effect [2]. This implies that cancer cells necessitate a greater quantity of glucose compared to healthy cells in order to sustain an adequate ATP supply for energy generation [3, 4]. Lactic acid fermentation, catalyzed by lactate dehydrogenase (LDH), is the predominant method by which numerous cancer cells produce ATP. This process involves the conversion of pyruvic acid into lactic acid as the end product. Also, aerobic glycolysis way was used to produce essential building blocks such as amino acids, lipids, and nucleotide synthesis [5]. Moreover, a number of studies have shown that LDH can be a powerful biomarker for early recognition of lung injury and severe COVID-19 cases. There were significant differences in LDH levels between patients and those without severe disease in early COVID-19 data [6].

Lactate Dehydrogenase (LDH) is a tetrameric enzyme critical for anaerobic respiration. Anaerobic respiration occurs when pyruvate is converted into lactate acid in the absence of oxygen. There are two known isoforms of LDH; LDHA and LDHB. The LDHB catalyzes the reversible conversion of lactate to pyruvate with the reduction of NAD + to NADH, while the LDHA catalyzes the reverse reaction. Scientists have discovered that LDHB is consistently expressed in different types of cancer cells, whereas LDHA may play an important role in tumor initiation since it is frequently overexpressed in cancer. The reduction of LDHA levels was associated with fewer cellular transformations and delayed tumor formation [7, 8].

A small molecule inhibitor that inhibits LDH proteins is required in cancer cells, lung tissue, and coronary arteries where high levels of LDH are present. There is a correlation between lymphocyte levels and LDH levels in the blood of COVID-19 patients, which is associated with the severity of the disease. Higher leukocyte count and LDH levels are indicative of an increased risk of mortality [9]. LDH levels in COVID-19 patients experiencing severe illness showed a significant up to sixfold elevation, which corresponded to a staggering 16-fold increase in mortality [6]. Granchi et al. provided a comprehensive review of inhibitors of lactate dehydrogenase (LDH) isoforms and their therapeutic potential. They described the various chemical classes of LDH inhibitors, including oxamic acid derivatives, pyrazole derivatives, quinoline derivatives, phenylpyruvic acid derivatives, and pyridine derivatives. They also highlighted recent advances in the development of LDH inhibitors and their potential applications in the treatment of cancer, infectious diseases, and other pathologies. Various factors can influence LDH inhibitory activity including the chemical structure of the inhibitor, the type and location of functional groups, the size and shape of the inhibitor molecule, and the interaction of the inhibitor with the active site of the LDH enzyme. It is noted that the potency of LDH inhibitors can be affected by the concentration of the enzyme, the pH and temperature of the reaction, and the presence of other substrates or cofactors. They also emphasized the importance of isoform selectivity in the design of LDH inhibitors, as different isoforms of LDH have different tissue distributions and may play different roles in disease [10]. Miskimins et al. propose that oxamate and phenformin exhibit synergistic anti-cancer effects by concurrently inhibiting complex I in mitochondria and LDH in the cytosol [11]. LDHA can be inhibited by galloflavin, a synthetic chemical that selectively binds to free enzymes without interfering with substrates or cofactors, and without causing any changes to mitochondrial respiration [12]. It has been attempted to treat COVID-19 with hydroxychloroquine, a medication commonly used to treat arthritis. Selenobenzene compounds exhibit LDHA inhibitory properties, according to Kim et al*.* [13]. New LDH inhibitors such as phthalimide and dibenzofuran selectively inhibit LDHA isoenzyme [14, 15]. Quinoline-3-sulfonamides, when compared to LDHB, exhibit higher selectivity for LDHA by competing with NADH [16]. Additionally, QSAR studies were performed on flavoalkaloids and flavonoids [17], quinoline-based derivatives [18], and tricyclic guanidine analogues of batzelladine K [19] as LDH inhibitors.

**Fig. 1:**
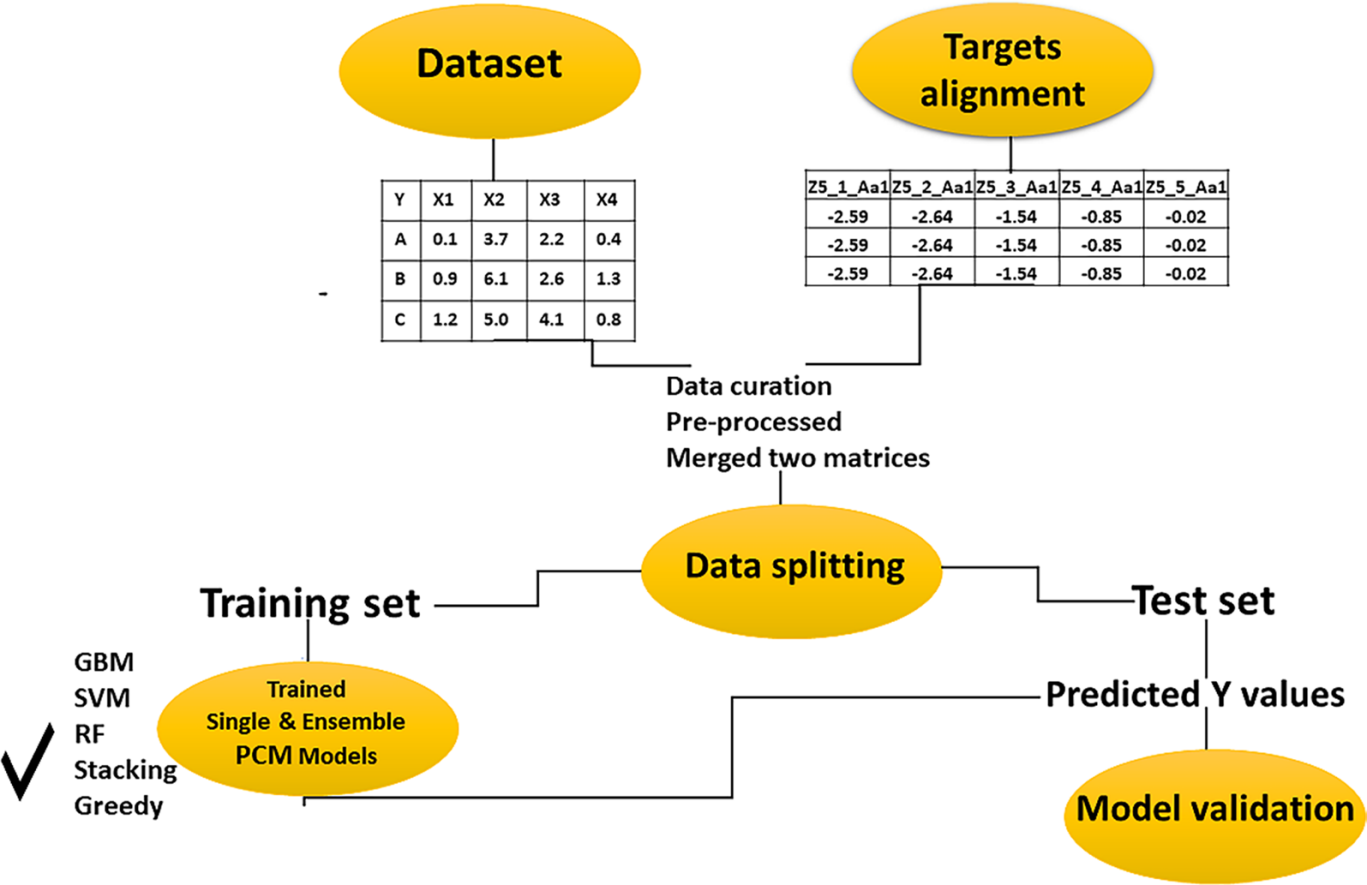
Flowchart process of proteochemometrics modeling

New strategies have been developed to improve the efficiency of the discovery and development of drugs due to the increased energy, time, and costs associated with this process. Drug discovery and optimization are becoming substantially more efficient by using computer-aided drug design (CADD). Drug design methods can be divided into three categories: ligand-based (LBDD); structure-based (SBDD); and system-based methods [20]. As with QSAR, LBDD is focused only on inferring relationships between structural and physicochemical attributes of ligands, as well as their corresponding biological properties [21]. SBDD elucidates the characteristics of current ligands or forecasts their attributes for novel ligands by utilizing experimental structures of protein targets, including receptors, enzymes, and proteins [22]. Systems-based drug development is based on genomic and proteomic information, their relationships, and how chemicals positively or adversely affect their expression. System-based drug design moves from a one drug–one target paradigm to a more systematic multidrug-multitarget paradigm, and the methods are inherently capable of unraveling complex networks of protein interactions with a library of compounds. Proteochemometrics modeling (PCM) is a systems-based approach that describes the interaction space of a series of compounds with a series of proteins [20, 23–25]. PCM combines descriptors of ligands and targets using machine learning algorithms to predict the bioactivity of compounds. This approach has the advantage of not requiring knowledge of the three-dimensional structure of the protein but instead relies on the amino acid sequence in order to generate descriptors. By integrating chemical and biological data, taking into account the available information in the model, it becomes possible to interpolate between the chemical and target spaces. This enables the prediction of the efficacy of (new) compounds on a range of (new) targets. Thus, it is possible to predict the bioactivity of new compounds on targets that have not yet been tested. Due to these features, PCM distinguishes itself from chemogenomics and QSAR by offering several advantages, including (i) the capability to incorporate bioactivity data from orthologous targets, (ii) the ability to forecast bioactivity for emerging viral mutations, and (iii) the potential for designing personalized medicine, such as tailored cancer therapies [26, 27]. PCM was introduced by Lapinsh et al*.* in 2001 [25], and since then, it has been successfully applied to a wide range of drug targets including proteases, kinases, cytochrome P450s, G protein-coupled receptors, and transport proteins [26, 28]. The advancement of robust machine learning techniques has resulted in their growing utilization for data-centric machine learning in Computer-Aided Drug Design (CADD) in recent times [29, 30]. It is likely that these methods will radically change the landscape of new molecules discovery and repurposing old drugs. Based on the PCM principles, we modeled the potency of 372 compounds on two isoforms of LDHA and LDHB. Various machine learning algorithms are used to train PCM models on public IC_50_ values from BindingDB. Ensemble modeling is then used to improve the performance models. An overview of the steps of the present study is shown in Fig. 1.

## Materials and methods

### Dataset

The dataset used to generate the PCM models was downloaded from the bindingBD [31] source. We accessed the data set by browsing by target name for LDH, which is the easiest way to access specific protein targets (www.bindingdb.org/bind/ByTargetNames.jsp). Once the data had been curated (data on human activity reported in IC_50_), 372 compounds were prepared to build models. To get more spread data points for biological activities (pIC_50_), the negative logarithmic transformation has been applied (− log IC_50_ × 10^–9^).

### Compound descriptors

The function StandardiseMolecules from the R package *camb* [32, 33], can be employed to standardize chemical structures in SMILES format according to the following procedure: (i) By comparing the structure of the entries, duplicates were removed from the dataset, (ii) removing all inorganic molecules, (iii) molecules were selected without requiring consideration of how many fluorines, chlorines, bromines or iodines were present in their structure or what their molecular mass was.

Morgan fingerprints [34] were calculated from Rdkit [35]. For the calculation of unhashed Morgan fingerprints, the dataset's compound substructures, with a maximum diameter of four bonds, were assigned distinct identifiers. The length of the fingerprints was specifically chosen as 512 in this case. Afterward, the substructures were transformed and organized into an unhashed array of counts. Physical descriptors were derived from the PaDEL [36] software by using the GeneratePadelDescriptors function in the R package *camb*.

### Protein descriptors

**Fig. 2:**
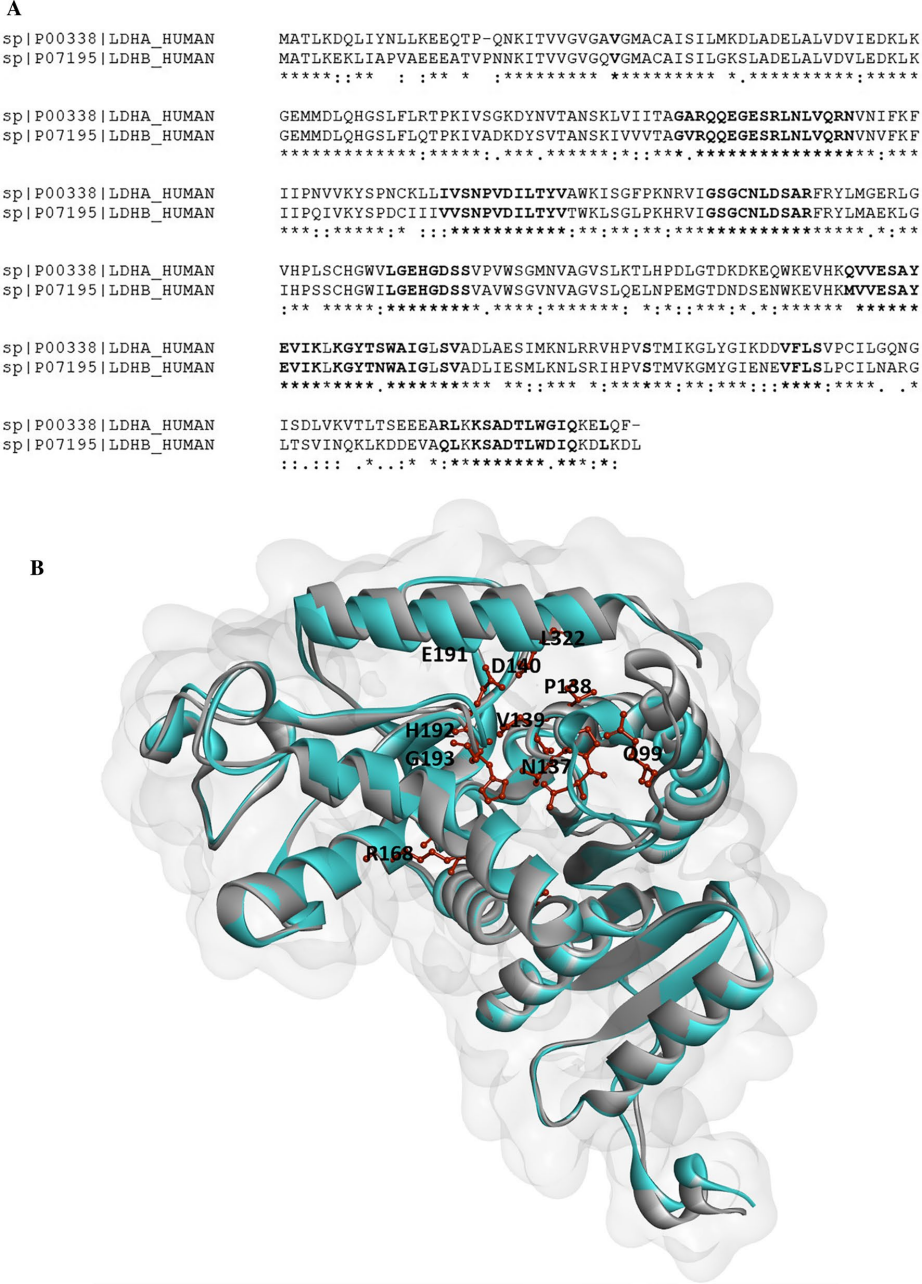
**A** Multiple sequence alignment of LDH homologous. Multiple sequence alignment of residues considered cavity amino acids is represented in bold. Conserved positions are marked by asterisks. **B** The superimposition structures of LDHA (PDB ID: 5W8J) and LDHB (PDB ID: 1I0Z)

For the alignment, the crystal structure of LDHA, identified by the 5W8J identifier, was used as a reference structure to identify the cavity of the enzyme. Clustal Omega web server [37] was used to align sequences with PDB ID of 1I0Z representing isoform LDHB. The conserved positions are shown by asterisks (Fig. 2a). A number of important residues involved in protein–ligand interactions have been identified by a previous X-ray study [38]. In order not to miss any of these critical residues, we used a cutoff 10 Å from the center of 2-{3-(3,4-difluorophenyl)-5-hydroxy-4-[(4-sulfamoylphenyl)methyl]-1H-pyrazol-1-yl}-1,3 thiazole-4-carboxylic acid inhibitor to determine ligand‐interacting residues [39]. Some of the residues located in the cavity and used for descriptor generation are shown in bold in Fig. 2b. The functions AADescs from the R package *camb* were used to calculate 5 Z-scales descriptors [40] for binding site amino acids of Lactate dehydrogenase.

Some of the residues located in the cavity and applied for descriptor generation are represented.

### Generation of PCM models by machine learning approaches

A matrix was constructed by concatenating compound and target descriptors and then compressing it using a preprocessing method. We removed highly correlated and near-zero variance descriptors using the functions RemoveHighlyCorrelatedFeatures (cut-off 0.95) and RemoveNearZeroVarianceFeatures (cut-off 30/1), respectively. Using the function PreProcess from the R package *camb*, the remaining descriptors were then centered to zero mean and scaled to unit variance. Based on stratified sampling according to bioactivity labels, the whole dataset was split into 70/30 training to test ratio randomly in *camb*. By applying Machine Learning models, descriptors were correlated with biological activities. We used gradient-boosting machines (GBMs) [41], Random Forests (RFs) [42], and Support Vector Machines (SVMs) [43] to train our models by using the GBM, RF, and SVMRadial methods respectively. Gradient boosting machines are an effective method for capturing complex functions with non-linear dependencies. SVM is a machine learning method used for classification and regression tasks. It utilizes kernel functions to transform data into a higher-dimensional space, enabling the identification of an optimal separating hyperplane that effectively distinguishes samples into distinct classes [44–46]. Random Forest is an approach that merges the forecasts of numerous unrelated decision trees. These trees are built using randomly selected independent vectors and are employed to make predictions for new inputs in classification or regression tasks. Decision trees, also referred to as regression trees, are constructed hierarchically, repeatedly dividing the dataset into different branches that maximize the information obtained from each division [47].

### Principal component analysis

PCA is a multivariate data analysis method that is commonly used to determine the similarities and differences between the sample and variables, thereby leading to data classification, outlier detection, and data reduction [48]. In PCA, multivariate data are transformed linearly into a smaller set of new orthogonal variables called principal components (PCs). The PCs contain considerable information regarding the original dataset. Samples are plotted using new axes and the resulting graphic is called Score Plot. The loading plot shows the relationship between the variables and how much each one affects the system. A PCA is performed on compound or protein descriptors using the *camb* function PCA. The output can be directly sent to PCAPlot, a tool that visualizes the first two principal components. This visualization includes the representation of the user-specified class, such as compound class or protein isoform, using shape and color.

### Model validation

The statistical robustness and good validation of models were corroborated based on criteria proposed by Golbraikh, Tropsha and Gramatica (Supplementary material). Moreover, The experimental error of the dependent variable (bioactivity values) is required to determine the maximum model performance [49]. In the absence of experimental uncertainty, the maximum ${R}_{0\, test}^{2}$ and minimum ${RMSE}_{test}$ distributions can be computed using the uncertainty in public bioactivity databases [50]. The model is likely to be over-optimistic if the metrics obtained were above the maximum values (for ${R}_{0 \,test}^{2}$) or below the minimum values (for ${RMSE}_{test}$) of the distribution. With the functions MaxPerf and MinPerf, you can compute the maximum and minimum ${R}_{0\, test}^{2}$ and ${RMSE}_{test}$ values. The methodology for calculating these parameters was explained in the Supplementary material.

### Ensemble modeling

Ensemble modeling techniques, such as greedy and stacking optimization, are applied using the caretEnsemble R package. This approach allows for the creation of ensemble models by combining multiple individual models, which have been shown to be more accurate and less prone to errors than standalone models [51]. In greedy optimization, using a linear combination of the prediction values from the input model, the cross-validated RMSE is optimized [52]. On a set of training data with the same fold composition, these models were trained. Following is a description of how each model is assigned a weight. In the beginning, all models had zero weight. After that, the weight of a specific model was incrementally increased by 1 whenever the normalized weight vector resulted in a closer alignment between the combined predictions from cross-validation and the observed pIC_50_ values. By default, n = 1000, thus repeating this step n times. In order to obtain a final weight vector, the resulting weight vector is normalized. In the process of model stacking, the predictions generated by the input models are utilized as training data for a meta-model. It is possible for this meta-model to have linear or non-linear characteristics [53]. If the algorithm selected is able to determine the importance of each input, each input is associated with an individual model, which in turn determines the relative contribution of each model to the prediction. Using this model ensemble, a test set (not used when the ensemble is constructed) can be used to compare the error metric (for example RMSE) between the ensemble and the single models.

## Results and discussion

### Analysis of PCM models

**Fig. 3:**
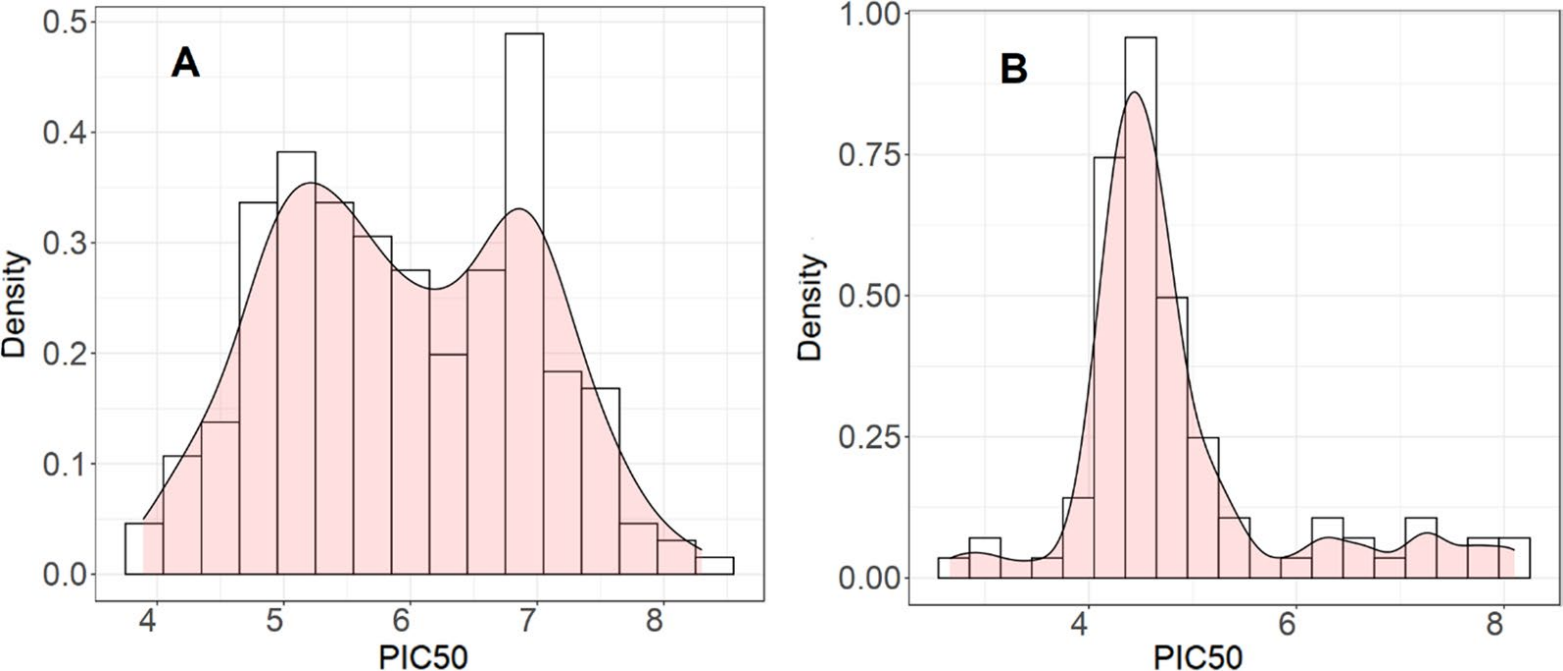
Density of the response variable for **A** LDHA, **B** LDHB

**Fig. 4:**
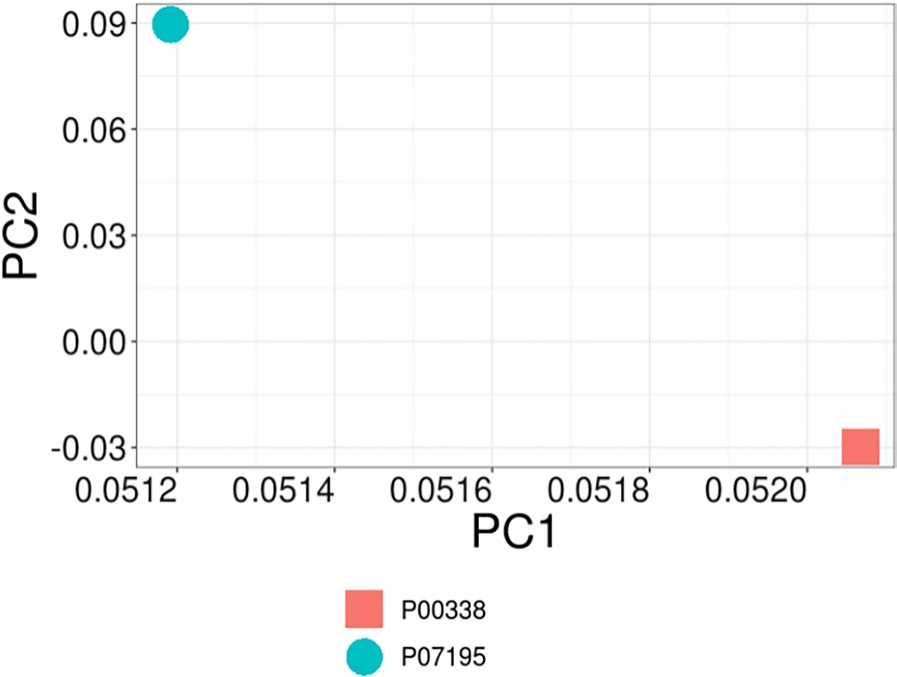
PCA analysis output from PCM. The function PCA was used to analyze the binding site amino acid descriptors (represented by 5 Z-scales). The first two principal components (PCs) accounted for over 76% of the variance, suggesting that the data primarily exhibits two main sources of variability. The LDHs can be observed to cluster into two distinct groups, corresponding to the isoenzymes LDHA and LDHB

Occasionally, in BindingDB for a compound there might be more than one bioactivity value. Duplicate pairs are removed with “remove_duplicates.R” and the mean bioactivity value is maintained. A total of 312 compounds remained after removing redundant pairs and were used for PCM modeling. Additional file 1: Table S1 shows their structures in SMILES format and pIC_50_. By using the function StandardiseMolecules with default parameters, it was possible to find a common representation for compound structures that kept all molecules, regardless of their molecular mass or the number of halogens they contained. As a visualization tool, histograms (DensityResponse) were used to explore the distribution of the response variable (Fig. 3). Figure 4 illustrates the PCA performed on the amino acid descriptors of the binding site for the two LDHs. This figure defines two distant clusters related to protein isoforms, LDHA and LDHB.

**Fig. 5:**
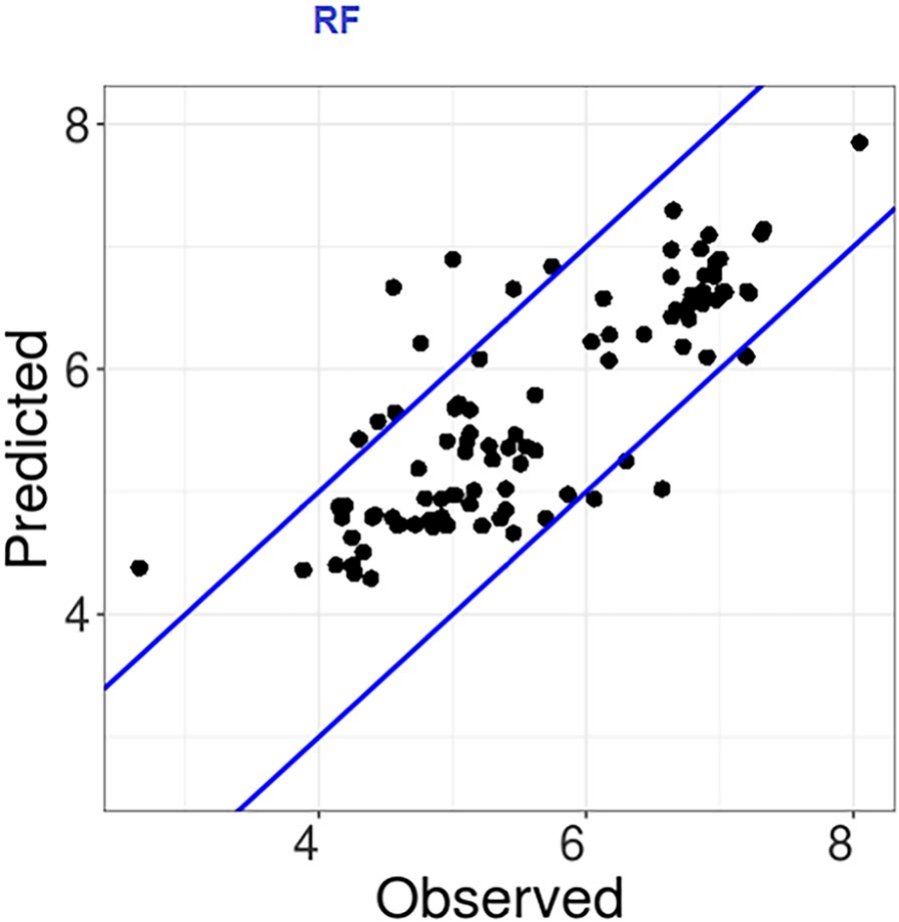
Observed against predicted values on the test set for RF model

SMILES format was used to represent molecules. We generated 1241 descriptors for ligands including 512 circular Morgan fingerprint descriptors using "MorganFPs" and RDkit, and 729 topological and physicochemical descriptors using the GeneratePadelDescriptors function and the PaDEL-Descriptor Java library. ImputeFeatures function was used to fill in the missing (NA" or "Inf”) descriptor values. 88 residues were selected in a radius of 10 Å centered on the ligand and calculated 5 Z-scales for these residues with the function AADescs (440 descriptors). Following these two filter steps, 286 descriptors were selected including 232 physicochemical descriptors, 24 Morgan fingerprint descriptors, and 30 Z-scales; (1) the function RemoveHighlyCorrelatedFeatures with a threshold value of 0.95 was used to remove descriptors with high correlation and redundant predictive signals, and (2) describing features with near-zero variance and therefore little predictive value were removed using RemoveNearZeroVarianceFeatures with a cut-off value of 30/1. The function PreProcess was used to scale all descriptors to have unit variance and zero mean prior to model training. To demonstrate the capabilities of *camb* for PCM modeling of compound properties, the dataset used comprised 312 data points including 218 for training and 94 for the test set. Three machine learning approaches including RF, SVM, and GBM for single PCM models were trained. The optimal value used for the RF model was mtry = 256. mtry is the number of features to consider at each split point. Optimize values of bandwidth of kernel function (σ = 0.01) and capacity parameter (C = 3) for SVM with radial basis function kernel were selected. Tuning parameters 'n.trees' (number of trees), 'interaction.depth' (maximum nodes per tree), and 'n.minobsinnode' (the minimum number of observations in terminal nodes) at GBM model were held constant at a value of 500, 25 and 20, respectively. 'Shrinkage' (learning rate) was considered at four values 0.04, 0.08, 0.12, and 0.16. RMSE was used to select the optimal model using the smallest value. The final values used for the model were n.trees = 500, interaction.depth = 25, shrinkage = 0.16 and n.minobsinnode = 20. As commonly recognized, a dependable model is indicated by R2 (or Q2) values that approach 1 and low RMSE or MAE values when predicting the test set or through cross-validation. In terms of statistical performance, the RF is better than the other PCM models (Table 1). With the 'Correlation Plot' function, we can see the correlation between observed and predicted values of test set for RF model as the best model (Fig. 5).

**Table 1:** Internal and external validation metrics for the single PCM models

Parameters	RF	SVM	GBM
${R}_{CV}^{2}$	0.89	0.54	0.92
${RMSE}_{CV}$	0.382	1.11	0.317
${R}_{test}^{2}$	0.62	0.29	0.57
${R}_{0\, test}^{2}$	0.62	0.09	0.55
$Q{}_{1}{}^{2}test$	0.62	0.07	0.53
$Q{}_{2}{}^{2}test$	0.62	0.07	0.54
$Q{}_{3}{}^{2}test$	0.67	0.18	0.60
${RMSE}_{test}$	0.65	1.01	0.71
$MAE$	0.46	0.84	0.54
$\frac{\left({R}_{test}^{2}-{R}_{0\, test}^{2}\right)}{{R}_{test}^{2}} <0.1$	0.0003	0.689	0.016
0.85 ≤ *k* ≤ 1.15	1.0005	1.030	0.983

**Fig. 6:**
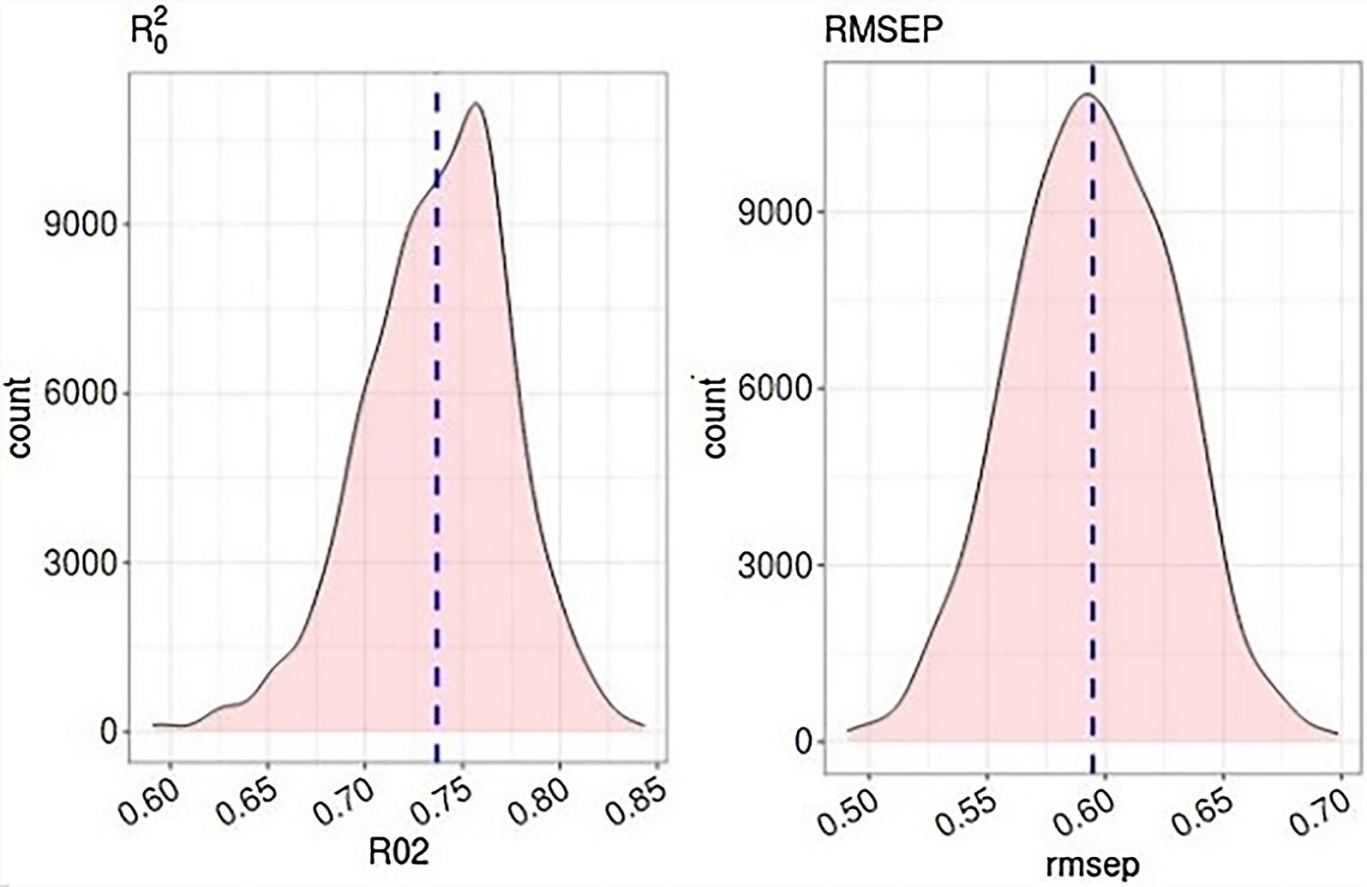
Distribution of theoretical ${R}_{0\, test}^{2}$ (**A**) and ${RMSE}_{test}$ values (**B**) for RF model

One way to improve model robustness and productivity is using of ensemble modeling (greedy and stacking optimization previously described). The file modelsEnsemble contains a list of models previously trained (RF, SVM and GBM). The ensemble models are created by greedy and stacking optimization methods after all models have been loaded. The function Validation is utilized to compute statistical metrics values for the test set. Except for svmRBF Best (Table 2), all model ensembles performed better predictive power on the test set than single PCM models. RF ensemble Best produced (bolded in Table 2) the highest ${R}_{0\, test}^{2}$ value, 0.65, and the lowest *RMSE*_*test*_ value, 0.62. "Best" ensembles are those trained on only the three most predictive RF, GBM, and SVM models. According to these findings, combining PCM models in more predictive model ensembles is associated with higher predictive power, although it may sometimes be marginal. On the other hands, Fig. 6 shows maximum and minimum distributions of ${R}_{0\, test}^{2}$ and *RMSE*_*test*_ values of 0.73 and 0.59, which are only marginally different from the ${R}_{0\, test}^{2}$ and *RMSE*_*test*_ of the RF best model with 0.65 and 0.62. Also, ensemble QSAR models were constructed in the *camb* package. According to statistical parameters calculated for the ensemble QSAR models in Additional file 1: Table S2 (Supplementary material), and comparing these values with the ensemble PCM models, we conclude that the PCM method was more suitable for predicting biological activities.

**Table 2:** Internal and external validation metrics for the ensemble PCM models

Parameters	GBM best	RF best	svmRBF Best	EN stacking SVMliner	EN stacking linear	EN greedy	EN stacking SVMRBF	EN stacking enet
${R}_{CV}^{2}$	0.90	**0.93**	0.54	0.90	0.92	–	0.54	0.65
${RMSE}_{CV}$	0.34	**0.29**	1.1	0.37	0.32	0.63	1.1	0.68
${R}_{test}^{2}$	0.63	**0.66**	0.4	0.65	0.65	0.65	0.64	0.65
${R}_{0\, test}^{2}$	0.62	**0.65**	0.11	0.64	0.64	0.64	0.63	0.64
$Q{}_{1}{}^{2}test$	0.61	**0.65**	0.09	0.64	0.64	0.63	0.62	0.64
$Q{}_{2}{}^{2}test$	0.61	**0.65**	0.09	0.64	0.64	0.64	0.64	0.64
$Q{}_{3}{}^{2}test$	0.68	**0.71**	0.24	0.7	0.70	0.7	0.69	0.7
${RMSE}_{test}$	0.66	**0.62**	1.01	0.63	0.63	0.63	0.65	0.63
$MAE$	0.505	**0.436**	0.495	0.492	0.486	0.485	0.509	0.486
$\frac{\left({R}_{test}^{2}-{R}_{0 \,test}^{2}\right)}{{R}_{test}^{2}} <0.1$	0.031	**0.0151**	0.725	0.0158	0.0158	0.0158	0.0156	0.0158
0.85 ≤ *k* ≤ 1.15	0.982	**0.982**	0.978	0.982	0.98	0.982	1.002	0.972

EN, elastic net

The RF ensemble Best model exhibited benefits in forecasting the activity (pIC50) of LDHA and LDHB inhibitors when compared to other proteochemometrics models. Nevertheless, it also had some limitations. The difference between internal validation (R^2^_cv_ = 0.93) and external validation (R^2^_test_ = 0.66) continues to persist. These outcomes suggest that the model's capability to predict the activity of novel compounds requires enhancement. This concern could potentially stem from a slight overfitting of the model. To tackle this issue, various approaches can be explored to enhance the model in the future. One possible strategy is to incorporate the regularization method during the construction of the fundamental model to alleviate overfitting. Furthermore, acquiring a more extensive dataset could aid in avoiding overfitting and enhancing the overall performance of the model. Additionally, investigating alternative feature selection methods may prove advantageous in reducing the model's complexity. The absence of feature selection methods in the *camb* is evident, highlighting the usefulness of such techniques as an effective strategy. Notwithstanding these limitations, the RF ensemble Best model remains applicable for predicting the activity of LDHA and LDHB inhibitors. Its favorable performance in internal validation indicates its potential effectiveness in these domains. With future enhancements and optimizations, its capabilities can be further improved.

**Fig. 7:**
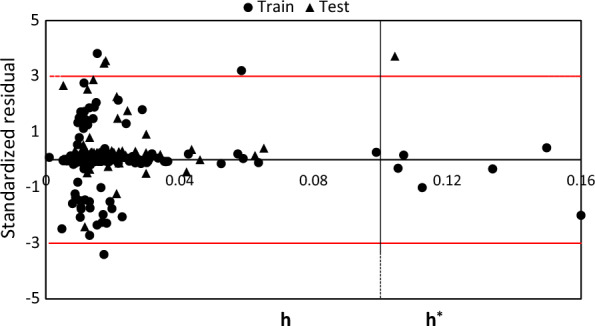
Plot of AD by the leverage distance method for RF-Best model

To enhance the validation of the RF-Best model, we employed the Application domains (AD) analysis. The ADs were established by employing the leverage distance method, which involved using Williams plots to compare the standardized residual (s) against the leverage (h) and to determine whether any influential chemicals or outliers were present. The chemicals that significantly influenced the model were identified by their hi value, which was greater than the threshold value h* (3p/n, where p and n represent the number of descriptors and chemicals, respectively). On the other hand, outliers were determined based on a standardized residual value that exceeded 3 units [54]. Based on the established model, we have identified six chemicals as influential compounds using the leverage distance method. These influential compounds have hi values larger than h* and standardized residuals |s| smaller than 3, as shown in Fig. 7. However, these compounds were not identified as outliers. On the other hand, three compounds in the training set and three compounds in the test set were identified as outliers, as their standardized residuals |s| were larger than 3.

### The important extracted descriptors in the PCM model

**Fig. 8:**
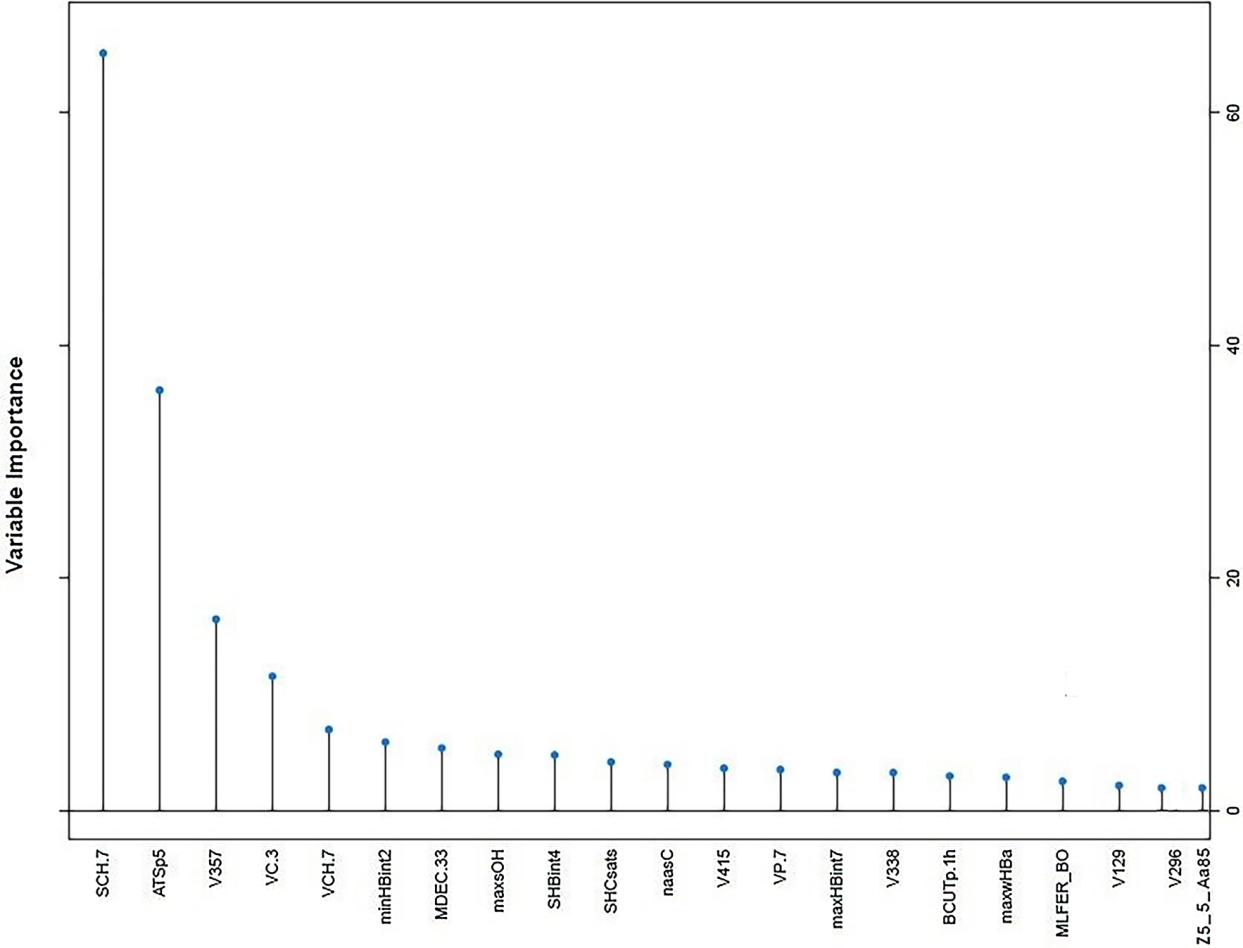
The 21 top descriptors selected in the RF-Best model

The top 21 most important descriptors in the RF-Best model were selected from the 286 input descriptors to build PCM models (Fig. 8).

It is important to note that these descriptors are related to compounds V357, V415, V338, V129, and V296. Descriptors are hashed Morgan fingerprints. The reason for abandoning hashed fingerprints was that they lack predefined substructural features and bit collision phenomena (the same bit is set by multiple patterns), which makes it nearly impossible to interpret specific fingerprint coordinates structurally [55]. The Chi index [56] descriptors are defined for the whole molecule. Chi index descriptors emphasize the variation of skeletal structure with particular attention to issues such as the degree of branching and the frequency of branching patterns, including branching adjacency and ring structure. These types of structure information are encoded by two types of Chi indices. The valence Chi indices account for atom valence states, whereas the simple Chi indices emphasize skeletal structure, independent of chemical elements. Chi indices are two-dimensional descriptors that include Chi chains, Chi clusters, and Chi path clusters. The autocorrelation descriptor is a topological descriptor that represents the degree of similarity between molecules and reflects the interdependence among atomic properties in a molecular graph [57]. Some previous studies used 2D autocorrelation descriptors to model the biological activities of synthetic chemicals [58–60]. The electrotopological state indices (E-states) were proposed by Kier and Hall for the characterization of atomic electronic and topological properties [61]. An E-state variable is assigned to each atom in the molecular graph, which encodes its intrinsic electronic state as perturbed by the electronic influence of all other atoms within the molecule within the context of its topological character. In this way, the E-state depends on the detailed structure of a molecule for a given atom (type). In linear free energy relations (LFERs), solvation parameters are used to describe the solvent–solute interactions [62]. In chemical and biochemical systems, Abraham's general solvation parameter model is one of the most useful approaches for analyzing and predicting free energies of partitioning. Among the independent descriptors in Abraham's equation, hydrogen-bond basicity appears in our PCM model. BCUT (Burden—CAS—University of Texas eigenvalues) metrics are extensions of parameters developed by Burden [63]. BCUT-values encode both substructural topological information (based on actual bonding or interatomic distances) and atomic properties relevant to ligand-receptor interactions (such as atomic charge, polarizability, H-bond donor and acceptor properties), and thus, can be used as chemistry-space metrics to assess pharmaceutical diversity. A BCUT descriptor related to polarizability in the PCM model gives important structural information. Based on Liu's suggestion, the molecular distance-edge (MDE) [64] can be calculated as follows for a molecule: each non-hydrogen atom of the molecule is considered a point and each chemical bond is considered an edge. The whole molecule is viewed as a topological graph. For example, using four different types of carbon atoms (secondary carbon in this study), MDE can discriminate between isomers of alkanes well. The MDE descriptors are practical and easy to use for modeling and can be correlated with many physical properties, such as Gibbs free energy and enthalpy as well as biological activity. On the other hand, the 21st important descriptor is a Z scale protein descriptor that has important value as well as the 20th descriptor, V296. Table 3 lists the two-dimensional top 21 selected descriptors in RF-Best PCM model with details. Inhibitory activation can be explained with Morgan fingerprints and topological structure descriptors, as shown in Table 3. The Chi indices can help us understand how the degree and frequency of branching patterns in the inhibitors affect their binding to the LDH enzyme. The E-state indices, on the other hand, can provide insights into the electronic properties of the inhibitors and how they interact with the electronic properties of the active site. The BCUT metrics can assess the chemical diversity of the inhibitors and their potential to interact with different types of receptors. Finally, the MDE descriptors provide information on the topological similarity between the inhibitors and the LDH enzyme, which can be used to predict their inhibitory activity. Overall, these descriptors provide a mechanistic understanding of the interaction between LDH and inhibitors by identifying the key structural and electronic factors that influence inhibitor binding. This understanding can be used to design more potent and selective LDH inhibitors, which could have significant therapeutic potential in the treatment of cancer and other diseases.

**Table 3:** The class, name and definition of descriptors selected in the RF-Best model

Descriptor Java class	Descriptor	Description
ECFP-hashfingerprint	V357, V415, V338 V129, V296	Generation of the fixed-length bit string
Chi chain	SCH.7	SCH-7—Simple chain, order 7
	VCH.7	VCH-7-Valence chain, order 7
Chi cluster	VC.3	Valence chain, order 3
Chi path cluster	VP.7	Valence path, order 7
Autocorrelation	ATSp5	Broto-Moreau autocorrelation—lag 5/weighted by polarizabilities
Atom type electrotopological state	minHBint2	Minimum E-State descriptors of strength for potential Hydrogen Bonds of path length 2
	SHBint4	Sum of E-State descriptors of strength for potential hydrogen bonds of path length 4
	maxHBint7	Maximum E-State descriptors of strength for potential Hydrogen Bonds of path length 7
	maxwHBa	Maximum E-States for weak Hydrogen Bond acceptors
	maxsOH	Maximum atom-type E-State: –OH
	SHCsats	Sum of atom-type H E-State: H on C sp3 bonded to saturated C
	naasC	Count of atom-type E-State::C:–
Molecular linear free energy relation	MLFER_BO	Overall or summation solute hydrogen bond basicity
BCUT	BCUTp.1 h	nlow highest polarizability weighted BCUTS
Molecular distance edge	MDEC	Molecular distance edge between all secondary carbons
5 Z scale protein descriptor	Z5_5_Aa85	Z5 for amino acid 85

Extended-connectivity fingerprints (ECFPs)

## Conclusion

PCM modeling was applied to the modeling of 312 compounds of LDHA and LDHB isoenzyme inhibitors by *camb* package. Combining chemical and target information in ensemble models improves the prediction of compound IC_50_ on human LDHA and LDHB compared with single models. LDH inhibitory activation is influenced by Morgan fingerprints and topological structure descriptors, according to the best model. Novel LDH inhibitors can be designed using the above information. In sum, PCM appears to be a suitable method for predicting compound activities by understanding how compounds interact with LDHA and LDHB. Further studies are needed to fully understand the biology of the LDH family in order to better predict the effects of compound interactions in cell-line models and in vivo.

## Supplementary Information

**Additional file 1.** Model validation ( Explained in detail and equations). **Table S1.** The SMILES format and pIc50 values for the train and test set. **Table S2.** Internal and external validation metrics for the ensemble QSAR models.

## Data Availability

The datasets used and/or analyzed during the current study are available from the corresponding author on reasonable request.
