# Supplementary material for: A study of the interaction space of two lactate dehydrogenase isoforms (LDHA and LDHB) and some of their inhibitors using proteochemometrics modeling
Source: BMC Chem. 2023 Jul 6;17(1):70. doi: 10.1186/s13065-023-00991-6 (PMC10324138; doi:10.1186/s13065-023-00991-6)
Supplement: Supplementary file 1 — Additional file 1. Model validation ( Explained in detail and equations). Table S1. The SMILES format and pIc50 values for the train and test set. Table S2. Internal and external validation metrics for the ensemble QSAR models. [file 13065_2023_991_MOESM1_ESM.docx]

**A study of the interaction space of two lactate dehydrogenase isoforms (LDHA & LDHB) and some of their inhibitors using proteochemometric modeling**

*Sedigheh Damavandi^1^, Fereshteh Shiri^2, *^, Abbasali Emamjomeh^1,3^, Somayeh Pirhadi^4^ and*

*Hamid Beyzaei^2^*

E-mail addresses:

F. Shiri^*^: [fereshteh.shiri@gmail.com](mailto:fereshteh.shiri@gmail.com) & Fereshteh.shiri@uoz.ac.ir

Tel./fax: +98 5431232186

*^1^Department of Bioinformatics, Laboratory of Computational Biotechnology and Bioinformatics (CBB Lab), University of Zabol, Zabol, Iran*

*^2^Department of Chemistry, Faculty of Science, University of Zabol, Zabol, Iran*

*^3^Department of Plant Breeding and Biotechnology (PBB), Faculty of Agriculture, University of Zabol, Zabol, Iran*

*^4^Medicinal and Natural Products Chemistry Research Center, Shiraz University of Medical Sciences, Shiraz, Iran*

**Model validation**

A PCM model should be robust and predictive if they are to be used to predict properties of new molecules. It is best to evaluate the predictability of a model by using it to predict the properties of new data not used in training. The models were subjected to external validation criteria according to the proposed test by Golbraikh, Tropsha and Gramatica[1-3]. We used both internal (cross-validation (Q^2^)) and external (external prediction ($R_{0}^{2}$)) validation approaches to determine the validity and predictability of the models. Leave-one-out cross-validated squared correlation coefficient (Q^2^), and N-fold cross-validation that show the model robustness are common internal validation methods. However, it has been shown clearly that the cross validated Q^2^ and test set R^2^ values often do not agree or correlate with each other. So, a reliable model has R^2^ (or Q^2^) values close to 1 and low RMSE or MAE values for prediction of the test set or from cross-validation. The formulas for the statistical metrics are as follows:

$RMSE=\sqrt{\frac{{(y-\tilde{y)}}^{2}}{N}}$ (1)

$$MAE= \frac{1}{n}\times\sum|y_{i}- \tilde{y}_{i}| (2)$$

$q_{CV}^{2}=1-\frac{\sum_{i=1}^{N} {(y_{i}-\tilde{y}_{i})}^{2}}{\sum_{i=1}^{N} {(y_{i}-\bar{y})}^{2}}$ (3)

$R_{0}^{2}=1-\frac{\sum_{i=1}^{N} {(y_{i}-\tilde{y}_{i}^{r^{0}})}^{2}}{\sum_{i=1}^{N} {(y_{i}-\bar{y})}^{2}}$ (4)

where $N$ represents the size of the training or test set, $y_{i}$the observed bioactivity values, $\tilde{y}_{i}$the predicted bioactivity values, and $\bar{y}$ the average values of the response variable for those datapoints included into either the training or the test set, and $\tilde{y}_{i}^{r^{0}}=s\tilde{y}$, with $s=\frac{\sum y_{i}\tilde{y}_{i}}{\sum\tilde{y}_{i}^{2}}$.

$R_{0 test}^{2}$ (the regression line must pass through the origin) value must be greater than 0.6 and $q^{2}>0.5$for a model to be considered predictive.

The formulas for the $Q_{1 test}^{2}, Q_{2 test}^{2} \mathrm{and} Q_{3 test}^{2}$ are as follows:

$Q_{1 test}^{2}\boldsymbol{=}1- \frac{\sum_{j=1}^{N_{test}} {(y_{j}-\tilde{y}_{j})}^{2}}{\sum_{j=1}^{N_{test}} {(y_{j}-\bar{y}_{tr})}^{2}}$ (5)

$Q_{2 test}^{2}\boldsymbol{=}1- \frac{\sum_{j=1}^{N_{test}} {(y_{j}-\tilde{y}_{j})}^{2}}{\sum_{j=1}^{N_{test}} {(y_{j}-\bar{y}_{test})}^{2}}$ (6)

$Q_{3 test}^{2}\boldsymbol{=}1-\frac{\frac{\left[ \sum_{j=1}^{N_{test}} \left( y_{\boldsymbol{j}}-\tilde{y}_{\boldsymbol{j}} \right)^{2} \right]}{N_{test}}}{\frac{\left[ \sum_{j=1}^{N_{tr}} \left( y_{\boldsymbol{j}}-\bar{y}_{tr} \right)^{2} \right]}{N_{tr}}} (7)$

In the above equations, $N_{test}, y_{j},\tilde{y}_{j} and \bar{y}_{test}$indicate the size, the observed, the predicted, and the average of the dependent variable for only those data points that make up the test set, respectively. $N_{tr} \mathrm{and}\bar{y}_{tr}$are the size and average values of the dependent variable for the datapoints that make up the training set. *j* defines the *j*th position in the training set.

Maximum model performance evaluates by $R_{0 test}^{2}$ and ${RMSE}_{test}$parameters. In the absence of experimental uncertainty, the maximum $R_{0 test}^{2}$ and minimum ${RMSE}_{test}$ distributions can be computed using the uncertainty in public bioactivity databases. The methodology for calculating maximum model performance parameters is as follow: From the experimental bioactivity values, a sample, A, randomly was selected with the same size as the external set. Then, sample B was calculated by adding a random noise with a mean and standard deviation equal to the experimental error to sample A. Next, the statistical metrics for A were calculated with respect to B. By calculating statistical metrics on 1,000 generations of random samples A and noisy samples B, a distribution of statistics was obtained for each dataset. As a result of getting these maximum and minimum values from the distribution, it was possible to validate the metrics values when evaluating the bioactivities predicted for the external sets. The model is likely to be over-optimistic if the metrics obtained were above the maximum values (for $R_{0 test}^{2}$) or below the minimum values (for ${RMSE}_{test}$) of the distribution.

| Table 1S. The SMILES format and pIc_50_ values for the train and test set | | |
| --- | --- | --- |
| Name | SMILES | pIc_50_ |
| Train set | | |
| m01 | Clc1ccccc1SC1C(=O)CC(NC1=O)(c1ccsc1)c1ccc(cc1)N1CCOCC1 | 8.3 |
| m02 | C[C@@H](OC(=O)Cc1ccc(Cl)c(SC2=C(O)CC(CC2=O)c2c(Cl)cccc2Cl)c1)c1ccccc1 | 8.22 |
| m03 | CC(C)Oc1ccc(Cl)c(C2CC(O)=C(Sc3cc(O)ccc3C | 7.82 |
| m04 | COc1ncc(-c2ccc3c(Nc4cc(cc(c4)C(O)=O)C4CCCC4)c(cnc3c2)S(=O)(=O)NC2CC2)c(OC)n1 | 7.8 |
| m05 | COc1ccc(Cl)c(SC2=C(O)CC(CC2=O)c2c(Cl)ccc(OC(C)C)c2Cl)c1 | 7.6 |
| m06 | CC(C)Oc1ccc(Cl)c(C2CC(O)=C(Sc3cc(O)ccc3Cl)C(=O)C2)c1Cl | 7.52 |
| m07 | CCOC(=O)Cc1ccc(Cl)c(SC2=C(O)CC(CC2=O)c2c(Cl)ccc(c2Cl)-c2ccccc2)c1 | 7.52 |
| m08 | COc1ccc(Cl)c(SC2=C(O)CC(CC2=O)c2c(Cl)ccc(NC3CCOCC3)c2Cl)c1 | 7.52 |
| m09 | COc1ccc(Cl)c(SC2=C(O)CC(CC2=O)c2c(Cl)ccc(c2Cl)-c2ccncc2)c1 | 7.46 |
| m10 | COC(=O)Cc1ccc(Cl)c(SC2=C(O)CC(CC2=O)c2c(Cl)cccc2Cl)c1 | 7.4 |
| m11 | Cc1c(Cc2ccc(cc2)S(N)(=O)=O)c(nn1-c1nc(cs1)C(O)=O)-c1ccccc1 | 7.38 |
| m12 | OC1=C(Sc2ccccc2Cl)C(=O)CC(C1)c1c(Cl)ccc(c1Cl)-c1ccccc1Cl | 7.22 |
| m13 | OC1=C(Sc2ccccc2Cl)C(=O)CC(C1)c1c(Cl)ccc(NC2CCOCC2)c1Cl | 7.22 |
| m14 | COc1ncc(c(OC)n1)-c1ccc2c(Nc3cc(cc(c3)C(O)=O)C(C)C(C)C)c(cnc2c1)C(N)=O | 7.2 |
| m15 | NS(=O)(=O)c1ccc(Oc2c(nn(-c3nc(cs3)C(O)=O)c2C2CC2)-c2ccccc2)cc1 | 7.19 |
| m16 | OC(=O)c1csc(n1)-n1nc(cc1O)-c1ccc(F)cc1F | 7.08 |
| m17 | OC1=C(Sc2ccccc2Cl)C(=O)CC(C1)c1c(Cl)ccc(c1Cl)-c1ccncc1 | 7.07 |
| m18 | C[C@H](OC(=O)Cc1ccc(Cl)c(SC2=C(O)CC(CC2=O)c2c(Cl)cccc2Cl)c1)c1ccccc1 | 7.05 |
| m19 | CC(C)Oc1ccc(Cl)c(C2CC(O)=C(Sc3ccccc3Cl)C(=O)C2)c1Cl | 7.05 |
| m20 | OC(=O)c1csc(n1)-n1nc(cc1O)-c1ccc(F)cc1 | 7.02 |
| m21 | OC(=O)c1csc(n1)-n1nc(cc1O)-c1cccc(c1)-c1ccccc1F | 7.02 |
| m22 | OC1=C(Sc2ccccc2Cl)C(=O)CC(C1)c1c(Cl)ccc(c1Cl)-c1ccccc1 | 7 |
| m23 | CC(=O)N1CCN(CC1)c1ccc(Cl)c(C2CC(O)=C(Sc3ccccc3Cl)C(=O)C2)c1Cl | 7 |
| m24 | OC(=O)c1csc(n1)-n1nc(cc1O)-c1ccc(Cl)c(Cl)c1 | 6.97 |
| m25 | OC1=C(Sc2ccccc2Cl)C(=O)CC(C1)c1c(Cl)ccc(c1Cl)-c1cccnc1 | 6.97 |
| m26 | OC(=O)c1csc(n1)-n1nc(cc1O)-c1cc(F)cc(F)c1 | 6.96 |
| m27 | NS(=O)(=O)c1ccc(Cc2c(nn(-c3nc(cs3)C(O)=O)c2C | 6.94 |
| m28 | OC1=C(Sc2ccccc2Cl)C(=O)NC(C1)(c1ccsc1)c1ccccc1 | 6.92 |
| m29 | CC(C)OC(=O)Cc1ccc(Cl)c(SC2=C(O)CC(CC2=O)c2c(Cl)cccc2Cl)c1 | 6.92 |
| m30 | OC1=C(Sc2cc(O)ccc2Cl)C(=O)CC(C1)c1c(Cl)cccc1Cl | 6.92 |
| m31 | NS(=O)(=O)c1ccc(Oc2cn(nc2-c2ccccc2)-c2nc(cs2)C(O)=O)cc1 | 6.91 |
| m32 | CCOC(=O)Cc1ccc(Cl)c(SC2=C(O)CC(CC2=O)c2c(Cl)cccc2Cl)c1 | 6.89 |
| m33 | OC(=O)c1csc(n1)-n1nc(cc1O)-c1ccccc1F | 6.87 |
| m34 | NS(=O)(=O)c1ccc(Cc2c(nn(-c3nc(cs3)C(O)=O)c2-c2ccccc2)-c2ccccc2)cc1 | 6.86 |
| m35 | OC(=O)c1csc(n1)-n1nc(cc1O)-c1ccccc1 | 6.84 |
| m36 | OC(=O)c1csc(n1)-n1nc(cc1O)-c1ccc(F)c(F)c1 | 6.82 |
| m37 | OC(=O)c1csc(n1)-n1nc(cc1O)-c1cccc(F)c1 | 6.79 |
| m38 | OC(=O)c1csc(n1)-n1nc(cc1O)-c1cccc(Cl)c1 | 6.77 |
| m39 | OC1=C(Sc2ccccc2Cl)C(=O)CC(C1)c1c(Cl)ccc(N2CCNCC2)c1Cl | 6.77 |
| m40 | CNc1ccc(Cl)c(SC2=C(O)CC(CC2=O)c2c(Cl)cccc2Cl)c1 | 6.77 |
| m41 | Nc1ccc2nc(SC3=C(O)CC(CC3=O)c3ccccc3)[nH]c2c1 | 6.74 |
| m42 | OC(=O)c1csc(n1)-n1nc(cc1O)-c1cccc(c1)C(F)(F)F | 6.72 |
| m43 | OC(=O)c1csc(n1)-n1nc(cc1O)-c1cccc2ccccc12 | 6.67 |
| m44 | COc1ccc(cc1)-c1cc(O)n(n1)-c1nc(cs1)C(O)=O | 6.64 |
| m45 | OC1=C(Sc2ccccc2Cl)C(=O)CC(C1)c1c(Cl)ccc(N2CCN(CC2)C2CC2)c1Cl | 6.64 |
| m46 | OC1=C(Sc2ccccc2Cl)C(=O)CC(C1)c1c(Cl)ccc(N2CCOCC2)c1Cl | 6.62 |
| m47 | OC(=O)c1csc(n1)-n1nc(cc1O)-c1cccc(c1)-c1ccccc1 | 6.58 |
| m48 | OC1=C(Sc2ncccn2)C(=O)CC(C1)c1ccccc1 | 6.57 |
| m49 | OC(=O)c1csc(n1)-n1nc(cc1O)-c1cccc(OC(F)(F)F)c1 | 6.52 |
| m50 | OCCNC(=O)Cc1ccc(Cl)c(SC2=C(O)CC(CC2=O)c2c(Cl)cccc2Cl)c1 | 6.52 |
| m51 | COc1ccc(Cl)c(SC2=C(O)CC(CC2=O)c2c(Cl)cccc2Cl)c1 | 6.43 |
| m52 | OC1=C(Sc2ccccc2Cl)C(=O)CC(C1)c1c(Cl)ccc(N2CCN(CC2)C2COC2)c1Cl | 6.43 |
| m53 | OC1=C(Sc2nc3ccccc3o2)C(=O)CC(C1)c1ccccc1 | 6.35 |
| m54 | COc1ccc(C | 6.34 |
| m55 | Cc1cc(C)nc(SC2=C(O)CC(CC2=O)c2ccccc2)n1 | 6.3 |
| m56 | Cc1nnc(SC2=C(O)CC(CC2=O)c2ccccc2)s1 | 6.24 |
| m57 | OC1=C(Sc2nc3ccc(Cl)cc3[nH]2)C(=O)CC(C1)c1ccccc1 | 6.19 |
| m58 | Nc1ccc(Cl)c(SC2=C(O)CC(CC2=O)c2c(Cl)cccc2Cl)c1 | 6.19 |
| m59 | CCC(Sc1nc(-c2ccc(Cl)cc2)c(C | 6.19 |
| m60 | OC(=O)c1csc(n1)-n1nc(cc1O)-c1ccncc1 | 6.17 |
| m61 | CC(Sc1nc(-c2ccc(Cl)c(F)c2)c(C | 6.15 |
| m62 | CC(Sc1nc(-c2ccc(Cl)c(Cl)c2)c(C | 6.12 |
| m63 | OC1=C(Sc2nc3ccccc3s2)C(=O)CC(C1)c1ccccc1 | 6.08 |
| m64 | OC1=C(Sc2ncc[nH]2)C(=O)CC(C1)c1ccccc1 | 6.06 |
| m65 | Nc1nnc(SC2=C(O)CC(CC2=O)c2ccccc2)s1 | 6 |
| m66 | OC(=O)c1csc(n1)-n1nc(cc1O)-c1ccccn1 | 5.99 |
| m67 | OC1=C(Sc2ccccc2Cl)C(=O)CC(C1)c1c(Cl)ccc(c1Cl)-c1cncnc1 | 5.96 |
| m68 | OC(=O)c1csc(n1)-n1nc(cc1O)C1CCCCC1 | 5.96 |
| m69 | OC1=C(Sc2ccccc2C | 5.8 |
| m70 | CNC(=O)Cc1ccc(Cl)c(SC2=C(O)CC(CC2=O)c2c(Cl)cccc2Cl)c1 | 5.74 |
| m71 | C[C@@H](Nc1nc(cnc1N)-c1cccc(c1)C(O)=O)c1ccc(Cl)cc1 | 5.7 |
| m72 | C[C@@H](Nc1nc(cnc1N)-c1cc(ccc1Cl)C(O)=O)c1ccccc1 | 5.7 |
| m73 | C[C@@H](Nc1nc(cnc1N)-c1cc(ccc1C)C(O)=O)c1ccccc1 | 5.7 |
| m74 | CC(C)c1ccc(cc1-c1cnc(N)c(N[C@H](C)c2ccccc2)n1)C(O)=O | 5.7 |
| m75 | COc1ccc(cc1-c1cnc(N)c(N[C@H](C)c2ccccc2)n1)C(O)=O | 5.7 |
| m76 | Cc1ccc2nc(SC3=C(O)CC(CC3=O)c3ccccc3)[nH]c2c1 | 5.68 |
| m77 | CCOc1cccc(c1)C1CC(O)=C(Sc2ccccc2Cl)C(=O)O1 | 5.66 |
| m78 | Cc1ccnc(SC2=C(O)CC(CC2=O)c2ccccc2)n1 | 5.64 |
| m79 | COc1cc(OCCCNC(=O)COc2cc(F)cc(c2)-c2ccc(cn2)C(O)=O)c(Cl)cc1NC(=O)CSc1ccc(cn1)C(O)=O | 5.62 |
| m80 | COc1cc(OC[C@@H](O)[C@@H](O)[C@H](O)[C@H](O)CNc2cc(F)cc(c2)-c2ccc(cn2)C(O)=O)c(Cl)cc1NC(=O)CSc1ccccn1 | 5.62 |
| m81 | OC1=C(Sc2ccccc2Cl)C(=O)CC(C1)c1c(Cl)cc(cc1Cl)N1CCOCC1 | 5.59 |
| m82 | C[C@@H](Nc1nc(cnc1N)-c1cccc(c1)C(O)=O)C1CCCCC1 | 5.52 |
| m83 | CCOC(=O)c1cnc(SC2=C(O)CC(CC2=O)c2ccccc2)nc1N | 5.51 |
| m84 | OC(=O)Cc1ccc(Cl)c(SC2=C(O)CC(CC2=O)c2c(Cl)cccc2Cl)c1 | 5.46 |
| m85 | OC1=C(Sc2ccccc2Cl)C(=O)OC(C1)c1ccccn1 | 5.46 |
| m86 | OC1=C(Sc2nncs2)C(=O)CC(C1)c1ccccc1 | 5.43 |
| m87 | CCOc1ccccc1C1CC(O)=C(Sc2ccccc2Cl)C(=O)O1 | 5.42 |
| m88 | OC1=C(Sc2ccccc2Cl)C(=O)OC(C1)c1cccc(Cl)c1 | 5.41 |
| m89 | C[C@@H](Nc1nc(cnc1N)-c1cc(C)cc(c1)C(O)=O)c1ccccc1 | 5.4 |
| m90 | C[C@@H](Nc1nc(cnc1N)-c1cccc(c1)C(O)=O)c1ccccc1 | 5.4 |
| m91 | OC1=C(Sc2ccccc2Cl)C(=O)NC(C1)c1ccccc1 | 5.4 |
| m92 | OC1=C(Sc2ccccc2Cl)C(=O)OC(C1)c1ccccc1 | 5.4 |
| m93 | OC1=C(Sc2ccccc2Cl)C(=O)NC(C1)c1ccccc1 | 5.4 |
| m94 | CC1(CC(O)=C(Sc2ccccc2Cl)C(=O)O1)c1ccccc1 | 5.39 |
| m95 | OC1=C(Sc2ccccc2Cl)C(=O)OC(C1)c1c(Cl)cccc1Cl | 5.3 |
| m96 | OC1=C(Sc2ccccc2Cl)C(=O)OC(C1)c1ccccc1Cl | 5.26 |
| m97 | OCC1(CC(O)=C(Sc2ccccc2Cl)C(=O)O1)c1ccccc1 | 5.25 |
| m98 | OC1=C(Sc2cccs2)C(=O)CC(C1)c1ccccc1 | 5.25 |
| m99 | Cc1cc(C)c(nc1SC1=C(O)CC(CC1=O)c1ccccc1)C | 5.25 |
| m100 | NC(=O)C1(CC(O)=C(Sc2ccccc2Cl)C(=O)O1)c1ccccc1 | 5.18 |
| m101 | Nc1cc(N)nc(SC2=C(O)CC(CC2=O)c2ccccc2)n1 | 5.17 |
| m102 | CCC(Sc1nc(-c2cccc(OC)c2)c(C | 5.13 |
| m103 | CSc1nnc(SC2=C(O)CC(CC2=O)c2ccccc2)s1 | 5.13 |
| m104 | CNC(=O)C1(CC(O)=C(Sc2ccccc2Cl)C(=O)O1)c1ccccc1 | 5.13 |
| m105 | OC1=C(Sc2ccccc2Cl)C(=O)OC(C1)c1cccc(c1)C | 5.02 |
| m106 | CC(=O)Nc1cc(nn1-c1nc(cs1)C(O)=O)-c1ccccc1 | 5.02 |
| m107 | C[C@@H](Nc1nc(cnc1N)-c1ccc(C(O)=O)c(O)c1)c1ccccc1 | 5 |
| m108 | CN(C)C(=O)Cc1ccc(Cl)c(SC2=C(O)CC(CC2=O)c2c(Cl)cccc2Cl)c1 | 5 |
| m109 | OC1=C(Oc2ccccc2Cl)C(=O)OC(C1)c1ccccc1 | 4.96 |
| m110 | OC1=C(Oc2ccccc2Cl)C(=O)NC(C1)c1ccccc1 | 4.96 |
| m111 | OC1=C(Sc2cc(Cl)ccc2Cl)C(=O)CC(C1)c1ccccc1 | 4.92 |
| m112 | Nc1ncc(nc1NCc1ccccc1)-c1cccc(c1)C(O)=O | 4.92 |
| m113 | NS(=O)(=O)c1ccc(NC(=O)CSc2nc(-c3ccccc3)c(C | 4.92 |
| m114 | CC(Sc1ncc(C | 4.9 |
| m115 | COc1cc(OC[C@@H](O)[C@@H](O)[C@H](O)[C@H](O)CNc2cc(F)cc(c2)-c2ccccn2)c(Cl)cc1NC(=O)CSc1ccc(cn1)C(O)=O | 4.89 |
| m116 | OC1=C(Sc2ccccc2Cl)C(=O)OC(C1)(C1CC1)c1ccccc1 | 4.89 |
| m117 | OC1=C(Nc2ccccc2Cl)C(=O)OC(C1)c1ccccc1 | 4.89 |
| m118 | C[C@@H](Nc1nc(cnc1N)-c1ccc(cc1)C(O)=O)c1ccccc1 | 4.85 |
| m119 | OC(=O)CCCc1ccc(cc1)-n1cc(CCCn2cc(C3=C(C(=O)NC3=O)c3c[nH]c4ccccc34)c3ccccc23)nn1 | 4.83 |
| m120 | C[C@@H](Nc1nc(cnc1N)-c1ccc(C)c(c1)C(O)=O)c1ccccc1 | 4.82 |
| *m121 | C[C@@H](Nc1nc(cnc1N)-n1cc(cn1)C(O)=O)c1ccccc1 | 4.82 |
| *m122 | Cc1cnc(SC2=C(O)CC(CC2=O)c2ccccc2)nc1O | 4.82 |
| *m123 | OC1=C(Sc2ccccc2Cl)C(=O)OC(C1)c1cccnc1 | 4.82 |
| *m124 | COc1cc(OC[C@H](O)[C@H](O)[C@@H](O)[C@@H](O)COc2cc(F)cc(c2)-c2ccc(cn2)C(O)=O)c(Cl)cc1NC(=O)CSc1ccc(cn1)C(O)=O | 4.82 |
| *m125 | Cc1cc(nn1-c1nc(cs1)C(O)=O)-c1ccccc1 | 4.81 |
| *m126 | OC1=C(Sc2ccccc2Cl)C(=O)OC(C1)c1ccc(Cl)cc1 | 4.8 |
| *m127 | Cc1cccc(C)c1C1CC(O)=C(Sc2ccccc2Cl)C(=O)O1 | 4.77 |
| *m128 | OC1=C(Sc2nc(O)cc(n2)C(F)(F)F)C(=O)CC(C1)c1ccccc1 | 4.77 |
| *m129 | NS(=O)(=O)N1CCN(Cc2cn(nc2-c2ccccc2)-c2nc(cs2)C(O)=O)CC1 | 4.76 |
| *m130 | OC1=C(Sc2nccs2)C(=O)CC(C1)c1ccccc1 | 4.74 |
| *m131 | C[C@@H](Nc1cc(cnc1N)-c1cccc(c1)C(O)=O)c1ccccc1 | 4.74 |
| *m132 | Cc1csc(SC2=C(O)CC(CC2=O)c2ccccc2)n1 | 4.72 |
| *m133 | Nc1ncc(nc1NCC1CCCC1)-c1cccc(c1)C(O)=O | 4.72 |
| *m134 | CN(C)C(=O)C1(CC(O)=C(Sc2ccccc2Cl)C(=O)O1)c1ccccc1 | 4.66 |
| *m135 | CC(C)c1ccc(NC(=O)CSc2nc(-c3ccc(Cl)cc3)c(C | 4.65 |
| *m136 | OC1=C(Sc2ccc(F)cc2Cl)C(=O)CC(C1)c1ccccc1 | 4.62 |
| *m137 | C[C@@H](Nc1nc(cnc1N)-c1ccnc(c1)C(O)=O)c1ccccc1 | 4.62 |
| *m138 | OC1=C(Nc2ccccc2Cl)C(=O)CC(C1)c1ccccc1 | 4.6 |
| *m139 | C[C@@H](Nc1cncc(n1)-c1cccc(c1)C(O)=O)c1ccccc1 | 4.6 |
| *m140 | NS(=O)(=O)c1ccc(Cc2c(nn(-c3nc(CO)cs3)c2C(F)(F)F)-c2ccccc2)cc1 | 4.59 |
| *m141 | OC1=C(Oc2ccccc2Cl)C(=O)CC(C1)c1ccccc1 | 4.59 |
| *m142 | COc1ccc(NC(=O)CSc2nc(-c3ccc(Cl)cc3)c(C | 4.58 |
| *m143 | Nc1cc(nn1-c1nc(cs1)C(O)=O)-c1ccccc1 | 4.57 |
| *m144 | Cc1ccc(NC(=O)CSc2nc(-c3ccc(Cl)cc3)c(C | 4.56 |
| *m145 | NC(=O)c1csc(n1)-n1nc(c(Cc2ccc(cc2)S(N)(=O)=O)c1O)-c1cccc(c1)-c1ccccc1 | 4.56 |
| *m146 | OC(=O)c1csc(n1)-n1nc(c(Cc2ccc(cc2)C(O)=O)c1O)-c1cccc(c1)-c1ccccc1 | 4.47 |
| *m147 | C[C@@H](Nc1nc(cnc1N)-c1cccc(C(O)=O)c1C)c1ccccc1 | 4.46 |
| *m148 | Cn1ccc(n1)C1CC(O)=C(Sc2ccccc2Cl)C(=O)O1 | 4.46 |
| *m149 | OC(=O)CCCCn1cc(CCCCn2cc(C3=C(C(=O)NC3=O)c3c[nH]c4ccccc34)c3ccccc23)nn1 | 4.44 |
| *m150 | OCc1ccccc1SC1=C(O)CC(CC1=O)c1ccccc1 | 4.44 |
| *m151 | OC(=O)c1ccc(cc1)-c1ccc(\C=N\NC(=O)CC | 4.43 |
| *m152 | OC(=O)c1cccc(c1)-c1ccc(\C=N\NC(=O)c2ccc3[nH]ccc3c2)o1 | 4.42 |
| *m153 | Cc1ccc(NC(=O)CSc2nc(-c3ccc(Cl)cc3)c(C | 4.41 |
| *m154 | OC(=O)c1cccc(c1)-c1ccc(\C=N\NC(=O)c2ccc3[nH]ncc3c2)o1 | 4.39 |
| *m155 | COc1cccc(c1)-c1nc(SCC(=O)Nc2ccc(cc2)S(N)(=O)=O)[nH]c(=O)c1C | 4.37 |
| *m156 | OC1=C(Sc2ccccc2Cl)C(=O)OC(C1)c1ccncc1 | 4.37 |
| *m157 | OC(=O)c1cccc(c1)-c1ccc(\C=N\NC(=O)c2cccnc2)o1 | 4.37 |
| *m158 | OCc1cccc(c1)C(=O)N\N=C\c1ccc(o1)-c1cccc(c1)C(O)=O | 4.34 |
| *m159 | OC(=O)C1(CC(O)=C(Sc2ccccc2Cl)C(=O)O1)c1ccccc1 | 4.34 |
| *m160 | Fc1ccc(NC(=O)CSc2nc(-c3ccc(Cl)cc3)c(C | 4.33 |
| *m161 | C[C@@H](Nc1nc(cnc1N)-c1csc(c1)C(O)=O)c1ccccc1 | 4.3 |
| *m162 | Cc1c(Cl)cccc1NC(=O)CSc1nc(-c2ccc(Cl)cc2)c(C | 4.3 |
| *m163 | OC1=C(Sc2ccccc2F)C(=O)CC(C1)c1ccccc1 | 4.28 |
| *m164 | OC1=C(Sc2ccccc2Br)C(=O)CC(C1)c1ccccc1 | 4.28 |
| *m165 | OC(=O)c1co[nH]c1=O | 4.27 |
| *m166 | Clc1ccc(NC(=O)CSc2nc(-c3ccc(Cl)cc3)c(C | 4.25 |
| *m167 | Clc1ccc(cc1)-c1nc(SCC(=O)Nc2cccc(Cl)c2)[nH]c(=O)c1C | 4.25 |
| *m168 | CNC(=O)CC1(CC(O)=C(Sc2ccccc2Cl)C(=O)O1)c1ccccc1 | 4.23 |
| *m169 | COc1cc(OCCCOCCCOc2cc(F)cc(c2)-c2ccc(cn2)C(O)=O)c(Cl)cc1NC(=O)CSc1ccc(cn1)C(O)=O | 4.23 |
| *m170 | OC1=C(Sc2cccc(Cl)c2Cl)C(=O)CC(C1)c1ccccc1 | 4.2 |
| *m171 | OC1=C(Nc2ccccc2Cl)C(=O)NC(C1)c1ccccc1 | 4.18 |
| *m172 | C[C@@H](Nc1nc(cnc1NS(C)(=O)=O)-c1cccc(c1)C(O)=O)c1ccccc1 | 4.15 |
| *m173 | OC1=C(Sc2ccccc2Cl)C(=O)OC(C1)c1cncnc1 | 4.14 |
| *m174 | CNc1ncc(nc1N[C@H](C)c1ccccc1)-c1cccc(c1)C(O)=O | 4.14 |
| *m175 | C[C@@H](Nc1nc(c(C)nc1N)-c1cccc(c1)C(O)=O)c1ccccc1 | 4.04 |
| *m176 | [Na+].NC(=O)C([O-])=O | 3.88 |
| *m177 | NC(=O)C1=CN(CCC1)[C@@H]1OC(COP([O-])(=O)OP([O-])(=O)OC[C@H]2O[C@H]([C@H](O)[C@@H]2O)n2cnc3c(N)ncnc23)[C@@H](O)[C@H]1O | 3.88 |
| *m178 | COc1cc(OCC(O)CO)c(Cl)cc1NC(=O)CSc1ccc(cn1)C(O)=O | 3.47 |
| *m179 | COc1ccc(Cl)cc1NC(=O)CSc1ccc(cn1)C(O)=O | 3.11 |
| *m180 | Nc1cc(F)cc(c1)-c1ccc(cn1)C(O)=O | 2.89 |
| *m181 | OC(=O)COc1cc(F)cc(c1)-c1ccc(cn1)C(O)=O | 2.66 |
| *m182 | NS(=O)(=O)c1ccc(Cc2c(CC3CC3)n(nc2-c2ccccc2)-c2nc(cs2)C(O)=O)cc1 | 8.1 |
| *m183 | NS(=O)(=O)c1ccc(Cc2c(nn(-c3nc(cs3)C(O)=O)c2C2CC2)-c2cccc(c2)-c2ccccc2)cc1 | 7.7 |
| m184 | NS(=O)(=O)c1ccc(Cc2c(nn(-c3nc(cs3)C(O)=O)c2C2CC2)-c2ccccc2)cc1 | 7.7 |
| m185 | NS(=O)(=O)c1ccc(Cc2c(CC3CC3)n(nc2-c2cccc(c2)-c2ccccc2)-c2nc(cs2)C(O)=O)cc1 | 7.57 |
| m186 | NS(=O)(=O)c1ccc(Cc2cn(nc2-c2cccc(c2)-c2ccccc2)-c2nc(cs2)C(O)=O)cc1 | 7.31 |
| m187 | NS(=O)(=O)c1ccc(Cc2c(nn(-c3nc(cs3)C(O)=O)c2C(F)(F)F)-c2cccc(c2)-c2ccccc2)cc1 | 7.21 |
| m188 | Nc1c(Cc2ccc(cc2)S(N)(=O)=O)c(nn1-c1nc(cs1)C(O)=O)-c1cccc(c1)-c1ccccc1 | 7.12 |
| m189 | NS(=O)(=O)c1ccc(Nc2cn(nc2-c2cccc(c2)-c2ccccc2)-c2nc(cs2)C(O)=O)cc1 | 7.11 |
| m190 | NS(=O)(=O)c1ccc(Nc2cn(nc2-c2ccccc2)-c2nc(cs2)C(O)=O)cc1 | 7.02 |
| m191 | NS(=O)(=O)c1ccc(Oc2c(nn(-c3nc(cs3)C(O)=O)c2C2CC2)-c2cccc(c2)-c2ccccc2)cc1 | 6.92 |
| m192 | CCOC(=O)Cc1ccc(Cl)c(SC2=C(O)CC(CC2=O)c2c(Cl)ccc(c2Cl)-c2ccccc2)c1 | 6.92 |
| m193 | NS(=O)(=O)c1ccc(Cc2cn(nc2-c2ccc(F)c(F)c2)-c2nc(cs2)C(O)=O)cc1 | 6.89 |
| m194 | CC(C)Oc1ccc(Cl)c(C2CC(O)=C(Sc3cc(O)ccc3C | 6.85 |
| m195 | CC(C)Oc1ccc(Cl)c(C2CC(O)=C(Sc3cc(O)ccc3Cl)C(=O)C2)c1Cl | 6.74 |
| m196 | NS(=O)(=O)c1ccc(Cc2cn(nc2-c2ccccc2)-c2nc(cs2)C(O)=O)cc1 | 6.69 |
| *m197 | COc1ncc(-c2ccc3c(Nc4cc(cc(c4)C(O)=O)C4CCCC4)c(cnc3c2)S(=O)(=O)NC2CC2)c(OC)n1 | 6.66 |
| m198 | COc1ccc(Cl)c(SC2=C(O)CC(CC2=O)c2c(Cl)ccc(c2Cl)-c2ccncc2)c1 | 6.64 |
| m199 | NS(=O)(=O)c1ccc(Cc2c(nn(-c3nc(cs3)C(O)=O)c2-c2cccnc2)-c2ccccc2)cc1 | 6.59 |
| m200 | COc1ccc(Cl)c(SC2=C(O)CC(CC2=O)c2c(Cl)ccc(OC(C)C)c2Cl)c1 | 6.39 |
| m201 | NS(=O)(=O)c1ccc(Cc2c(O)n(nc2-c2cccc(c2)-c2ccccc2)-c2nc(cs2)C(O)=O)cc1 | 6.37 |
| m202 | COc1ccc(Cl)c(SC2=C(O)CC(CC2=O)c2c(Cl)ccc(NC3CCOCC3)c2Cl)c1 | 6.31 |
| m203 | NS(=O)(=O)c1ccc(Cc2c(O)n(nc2-c2ccc(F)c(F)c2)-c2nc(cs2)C(O)=O)cc1 | 6.14 |
| m204 | OC1=C(Sc2ccccc2Cl)C(=O)NC(C1)(c1ccsc1)c1ccccc1 | 6.13 |
| m205 | OC1=C(Sc2ccccc2C | 6.07 |
| m206 | NS(=O)(=O)c1ccc(Cc2c(O)n(nc2-c2cccc(c2)-c2ccccc2F)-c2nc(cs2)C(O)=O)cc1 | 6.04 |
| m207 | OC1=C(Sc2ccccc2Cl)C(=O)CC(C1)c1c(F)cccc1F | 5.89 |
| m208 | NS(=O)(=O)c1ccc(Cc2c(O)n(nc2-c2cccc(Br)c2)-c2nc(cs2)C(O)=O)cc1 | 5.82 |
| m209 | Cc1cccc(C)c1C1CC(O)=C(Sc2ccccc2Cl)C(=O)C1 | 5.72 |
| m210 | OC1=C(Sc2ccccc2C | 5.7 |
| m211 | CC(Sc1nc(-c2ccc(Cl)c(F)c2)c(C | 5.64 |
| m212 | COc1ccc(C | 5.62 |
| m213 | CCC(Sc1nc(-c2ccc(Cl)cc2)c(C | 5.62 |
| m214 | OC1=C(Sc2ccccc2C | 5.55 |
| m215 | NS(=O)(=O)c1ccc(Cc2c(nn(-c3nc(cs3)C(O)=O)c2-c2ccncc2)-c2ccccc2)cc1 | 5.55 |
| m216 | CC(Sc1nc(-c2ccc(Cl)cc2)c(C | 5.52 |
| *m217 | OC1=C(Sc2ccccc2Cl)C(=O)CC(C1)c1c(Cl)cccc1Br | 5.47 |
| *m218 | CC(Sc1nc(-c2ccc(Cl)c(Cl)c2)c(C | 5.43 |
| Test set | | |
| m219 | OC1=C(Sc2ccccc2C | 5.31 |
| m220 | C[C@@H](Nc1nc(cnc1N)-c1cccc(c1)C(O)=O)c1ccc(Cl)cc1 | 5.3 |
| m221 | OC1=C(Sc2ccccc2Cl)C(=O)CC(C1)c1c(F)cccc1Cl | 5.28 |
| m222 | C[C@@H](Nc1nc(cnc1N)-c1cc(ccc1C)C(O)=O)c1ccccc1 | 5.22 |
| *m223 | OC1=C(Sc2ccccc2C | 5.18 |
| *m224 | COc1ccc(cc1)C1CC(O)=C(Sc2ccccc2Cl)C(=O)C1 | 5.16 |
| m225 | C[C@@H](Nc1nc(cnc1N)-c1cc(C)cc(c1)C(O)=O)c1ccccc1 | 5.15 |
| m226 | CC(C)c1ccc(cc1-c1cnc(N)c(N[C@H](C)c2ccccc2)n1)C(O)=O | 5.15 |
| m227 | COc1ccc(cc1-c1cnc(N)c(N[C@H](C)c2ccccc2)n1)C(O)=O | 5.15 |
| m228 | OC1=C(Sc2ccccc2Cl)C(=O)CC(C1)c1c(cccc1C(F)(F)F)C(F)(F)F | 5.13 |
| *m229 | CCC(Sc1nc(-c2cccc(OC)c2)c(C | 5.11 |
| m230 | Cc1cccc(C)c1C1CC(O)=C(Sc2ccccc2C | 5.11 |
| m231 | C[C@@H](Nc1nc(cnc1N)-c1cc(ccc1Cl)C(O)=O)c1ccccc1 | 5.1 |
| *m232 | Cc1nc2ccc(NC(=O)CCNC(=O)CCCc3ccc(CC(C(O)=O)C(O)=O)cc3)cc2s1 | 5.04 |
| m233 | NC(=O)C1=CN(CCC1)[C@@H]1OC(COP([O-])(=O)OP([O-])(=O)OC[C@H]2O[C@H]([C@H](O)[C@@H]2O)n2cnc3c(N)ncnc23)[C@@H](O)[C@H]1O | 5.03 |
| m234 | OC1=C(Sc2ccccc2C | 4.96 |
| m235 | NS(=O)(=O)c1ccc(NC(=O)CSc2nc(-c3ccc(Cl)cc3)c(C | 4.95 |
| m236 | C[C@@H](Nc1nc(cnc1N)-c1cccc(c1)C(O)=O)c1ccccc1 | 4.92 |
| m237 | OC1=C(Sc2ccccc2Cl)C(=O)CC(C1)c1ccccc1Cl | 4.89 |
| m238 | C[C@@H](Nc1nc(cnc1N)-n1cc(cn1)C(O)=O)c1ccccc1 | 4.82 |
| m239 | OC1=C(Sc2ccccc2Cl)C(=O)CC(C1)c1c(Br)cccc1Br | 4.82 |
| m240 | C[C@@H](Nc1nc(cnc1N)-c1ccc(cc1)C(O)=O)c1ccccc1 | 4.8 |
| m241 | C[C@@H](Nc1nc(cnc1N)-c1cccc(c1)C(O)=O)C1CCCCC1 | 4.8 |
| m242 | C[C@@H](Nc1nc(cnc1N)-c1ccc(C(O)=O)c(O)c1)c1ccccc1 | 4.8 |
| m243 | OC1=C(Sc2ccccc2Cl)C(=O)NC(C1)c1ccccc1 | 4.74 |
| m244 | C[C@@H](Nc1cncc(n1)-c1cccc(c1)C(O)=O)c1ccccc1 | 4.64 |
| m245 | OC1=C(Sc2ccccc2Cl)C(=O)CC(C1)c1cccc(Cl)c1 | 4.57 |
| *m246 | Nc1ncc(nc1NCc1ccccc1)-c1cccc(c1)C(O)=O | 4.55 |
| *m247 | OC1=C(Sc2ccccc2Cl)C(=O)CC(C1)c1ccc(Cl)cc1 | 4.43 |
| m248 | C[C@@H](Nc1nc(cnc1N)-c1ccc(C)c(c1)C(O)=O)c1ccccc1 | 4.4 |
| m249 | NS(=O)(=O)c1ccc(NC(=O)CSc2nc(-c3ccccc3)c(C | 4.37 |
| m250 | C[C@@H](Nc1nc(cnc1N)-c1cccc(C(O)=O)c1C)c1ccccc1 | 4.36 |
| m251 | C[C@@H](Nc1nc(cnc1N)-c1ccnc(c1)C(O)=O)c1ccccc1 | 4.32 |
| m252 | OC1=C(Sc2ccccc2Cl)C(=O)CC(C1)c1c(Cl)cccc1C(F)(F)F | 4.3 |
| m253 | OC(=O)c1co[nH]c1=O | 4.27 |
| m254 | C[C@@H](Nc1cc(cnc1N)-c1cccc(c1)C(O)=O)c1ccccc1 | 4.25 |
| *m255 | COc1cccc(c1)-c1nc(SCC(=O)Nc2ccc(cc2)S(N)(=O)=O)[nH]c(=O)c1C | 4.25 |
| *m256 | OC(=O)c1no[nH]c1=O | 4.14 |
| *m257 | Nc1ncc(nc1NCC1CCCC1)-c1cccc(c1)C(O)=O | 4.1 |
| m258 | C[C@@H](Nc1nc(cnc1N)-c1cccc(c1)C(N)=O)c1ccccc1 | 4.04 |
| *m259 | Cc1ccc(NC(=O)CSc2nc(-c3ccc(Cl)cc3)c(C | 4.03 |
| m260 | CC(C)c1ccc(NC(=O)CSc2nc(-c3ccc(Cl)cc3)c(C | 4.01 |
| m261 | OC(=O)c1ns[nH]c1=O | 4.17 |
| m262 | OC1=C(Sc2ccccc2Cl)C(=O)CC(C1)c1c(Cl)cccc1Cl | 6.06 |
| *m263 | OC1=C(Sc2ccccc2Cl)C(=O)CC(C1)c1ccccc1 | 5.36 |
| *m264 | OC1=C(Sc2ccccc2[N+]([O-])=O)C(=O)CC(C1)c1ccccc1 | 5.86 |
| m265 | OC(=O)c1csc(n1)-n1nc(cc1C(F)(F)F)-c1ccccc1 | 4.6 |
| m266 | COc1cc(OC[C@@H](O)[C@@H](O)[C@H](O)[C@H](O)CNc2cc(F)cc(c2)-c2ccc(cn2)C(O)=O)c(Cl)cc1NC(=O)CSc1ccc(cn1)C(O)=O | 6.92 |
| m267 | COc1cc(OC[C@@H](O)[C@@H](O)[C@H](O)[C@H](O)COc2cc(F)cc(c2)-c2ccc(cn2)C(O)=O)c(Cl)cc1NC(=O)CSc1ccc(cn1)C(O)=O | 6.5 |
| m268 | NS(=O)(=O)c1ccc(NC(=O)CSc2nc(-c3ccc(Cl)cc3)c(C | 5.39 |
| *m269 | CC(Sc1nc(-c2ccc(Cl)cc2)c(C | 6.32 |
| *m270 | C[C@@H](Nc1nc(cnc1N)-c1cc(ccc1C)C(O)=O)c1ccc(Cl)cc1 | 6.3 |
| m271 | COc1ncc(-c2ccc3c(Nc4cc(Oc5cc(F)cc(F)c5)cc(c4)C(O)=O)c(cnc3c2)S(=O)(=O)NC2CC2)c(OC)n1 | 8 |
| m272 | COC(=O)c1cc2c(cc(cc2n1O)-c1ccccc1)C(F)(F)F | 4.91 |
| m273 | OC1=C(Sc2ccccc2Cl)C(=O)OC2(CCOc3ccccc23)C1 | 5.02 |
| m274 | OC1=C(Sc2ccccc2Cl)C(=O)OC2(CCCc3ccccc23)C1 | 5.17 |
| m275 | OC1=C(Sc2ccccc2Cl)C(=O)OC2(CCc3ccccc23)C1 | 6.01 |
| m276 | OC1=C(Sc2ccccc2Cl)C(=O)OC2(CCc3c2cccc3Br)C1 | 6.07 |
| m277 | OC1=C(Sc2ccccc2Cl)C(=O)OC2(CCc3c2cccc3-c2ccccc2)C1 | 6.87 |
| m278 | COc1cc(cc(OC)c1OC)C(=O)N\N=C\c1cc(co1)-c1cccc(c1)C(O)=O | 4.39 |
| *m279 | COc1cc(cc(OC)c1OC)C(=O)N\N=C\c1ccc(s1)-c1cccc(c1)C(O)=O | 4.18 |
| m280 | OC(=O)c1cccc(c1)-c1ccc(\C=N\NC(=O)CC | 4.13 |
| m281 | COc1cc(cc(OC)c1OC)C(=O)N\N=C\c1ccc(o1)-c1cccc(c1)C(O)=O | 4.4 |
| m282 | COc1cc(cc(OC)c1OC)C(=O)N\N=C\c1ccc(o1)-c1ccc(cc1)C(O)=O | 3.89 |
| *m283 | NS(=O)(=O)c1ccc(Cc2c(CC3CC3)n(nc2-c2ccccc2)-c2nc(cs2)C(O)=O)cc1 | 8.05 |
| m284 | NS(=O)(=O)c1ccc(Cc2c(O)n(nc2-c2cccc(c2)-c2ccccc2)-c2nc(cs2)C(O)=O)cc1 | 6.46 |
| m285 | NS(=O)(=O)c1ccc(Cc2c(nn(-c3nc(cs3)C(O)=O)c2-c2cccnc2)-c2ccccc2)cc1 | 6.46 |
| m286 | NS(=O)(=O)c1ccc(Cc2cn(nc2-c2ccc(F)c(F)c2)-c2nc(cs2)C(O)=O)cc1 | 7.02 |
| m287 | Nc1c(Cc2ccc(cc2)S(N)(=O)=O)c(nn1-c1nc(cs1)C(O)=O)-c1cccc(c1)-c1ccccc1 | 7.19 |
| *m288 | NS(=O)(=O)c1ccc(Nc2cn(nc2-c2cccc(c2)-c2ccccc2)-c2nc(cs2)C(O)=O)cc1 | 7.24 |
| *m289 | NS(=O)(=O)c1ccc(Nc2cn(nc2-c2ccccc2)-c2nc(cs2)C(O)=O)cc1 | 7.04 |
| *m290 | NS(=O)(=O)c1ccc(Cc2c(O)n(nc2-c2ccc(F)c(F)c2)-c2nc(cs2)C(O)=O)cc1 | 6.17 |
| *m291 | OC(=O)c1csc(n1)-n1nc(cc1C(F)(F)F)-c1ccc(Cl)cc1 | 4.57 |
| m292 | NS(=O)(=O)c1ccc(Cc2cn(nc2-c2cccc(c2)-c2ccccc2)-c2nc(cs2)C(O)=O)cc1 | 7.42 |
| m293 | OC(=O)c1csc(n1)-n1nc(cc1C(F)(F)F)-c1ccc(F)cc1 | 4.56 |
| m294 | NS(=O)(=O)c1ccc(Cc2c(CC3CC3)n(nc2-c2cccc(c2)-c2ccccc2)-c2nc(cs2)C(O)=O)cc1 | 7.49 |
| m295 | NS(=O)(=O)c1ccc(Oc2c(nn(-c3nc(cs3)C(O)=O)c2C2CC2)-c2cccc(c2)-c2ccccc2)cc1 | 6.96 |
| m296 | NS(=O)(=O)c1ccc(Cc2c(O)n(nc2-c2cccc(Br)c2)-c2nc(cs2)C(O)=O)cc1 | 5.63 |
| *m297 | NS(=O)(=O)c1ccc(Cc2c(nn(-c3nc(cs3)C(O)=O)c2C2CC2)-c2ccccc2)cc1 | 7.72 |
| m298 | NS(=O)(=O)c1ccc(Cc2cn(nc2-c2ccccc2)-c2nc(cs2)C(O)=O)cc1 | 6.75 |
| *m299 | NS(=O)(=O)c1ccc(Cc2c(nn(-c3nc(cs3)C(O)=O)c2-c2ccncc2)-c2ccccc2)cc1 | 5.21 |
| m300 | NS(=O)(=O)c1ccc(Cc2c(O)n(nc2-c2cccc(c2)-c2ccccc2F)-c2nc(cs2)C(O)=O)cc1 | 6.12 |
| m301 | NS(=O)(=O)c1ccc(Cc2c(nn(-c3nc(cs3)C(O)=O)c2C2CC2)-c2cccc(c2)-c2ccccc2)cc1 | 7.57 |
| *m302 | NS(=O)(=O)c1ccc(Cc2c(nn(-c3nc(cs3)C(O)=O)c2C(F)(F)F)-c2cccc(c2)-c2ccccc2)cc1 | 7.28 |
| m303 | OC1=C(Sc2ccccc2Cl)C(=O)N[C@@](C1)(c1ccsc1)c1ccc(cc1)N1CCOCC1 | 7.09 |
| m304 | Cc1nc2ccc(NC(=O)CCNC(=O)CCCc3ccc(CC(C(O)=O)C(O)=O)cc3)cc2s1 | 5.93 |
| m305 | NC(=O)C(O)=O | 4.24 |
| *m306 | OC(=O)c1ns[nH]c1=O | 5.05 |
| *m307 | OC1=C(Sc2ccccc2Cl)C(=O)CC(C1)c1c(Cl)cccc1Cl | 5.05 |
| m308 | OC1=C(Sc2ccccc2Cl)C(=O)CC(C1)c1ccccc1 | 4.72 |
| m309 | OC1=C(Sc2ccccc2[N+]([O-])=O)C(=O)CC(C1)c1ccccc1 | 4.92 |
| *m310 | COc1ncc(-c2ccc3c(Nc4cc(Oc5cc(F)cc(F)c5)cc(c4)C(O)=O)c(cnc3c2)S(=O)(=O)NC2CC2)c(OC)n1 | 6.65 |
| *m311 | OC1=C(Sc2ccccc2Cl)C(=O)N[C@@](C1)(c1ccsc1)c1ccc(cc1)N1CCOCC1 | 7.33 |
| *m312 | NC(=O)C(O)=O | 4.47 |

*=Inhibitors for LDHB

| Table 2S. Internal and external validation metrics for the ensemble QSAR models | | | | | | | | |
| --- | --- | --- | --- | --- | --- | --- | --- | --- |
| Parameters | GBM Best | RF  Best | svmRBF Best | EN stacking  SVMliner | EN stacking  linear | EN  greedy | EN stacking  SVMRBF | EN stacking  enet |
| $R_{test}^{2}$ | 0.58 | 0.58 | 0.3 | 0.59 | 0.59 | 0.59 | 0.59 | 0.60 |
| $R_{0 test}^{2}$ | 0.57 | 0.57 | 0.11 | 0.58 | 0.58 | 0.58 | 0.59 | 0.58 |
| $Q{}_{1}^{2}{test}$ | 0.55 | 0.56 | 0.1 | 0.56 | 0.57 | 0.56 | 0.58 | 0.57 |
| $Q{}_{2}^{2}{test}$ | 0.55 | 0.56 | 0.1 | 0.56 | 0.57 | 0.56 | 0.58 | 0.57 |
| $Q{}_{3}^{2}{test}$ | 0.61 | 0.61 | 0.25 | 0.60 | 0.60 | 0.59 | 0.61 | 0.60 |
| ${RMSE}_{test}$ | 0.71 | 0.70 | 1.002 | 0.70 | 0.69 | 0.7 | 0.69 | 0.69 |
| $MAE$ | 0.53 | 0.50 | 0.83 | 0.52 | 0.51 | 0.52 | 0.51 | 0.51 |
| $\frac{\left( R_{test}^{2}-R_{0 test}^{2} \right)}{R_{test}^{2}} <0.1$ | 0.0172 | 0.172 | 0.63 | 0.169 | 0.033 | 0.033 | 0.166 | 0.033 |
| 0.85 ≤ *k* ≤ 1.15 | 1.005 | 1.011 | 0.95 | 1.007 | 1.005 | 1.006 | 1.006 | 1.005 |
| EN: Elastic Net | | | | | | | | |

[1] A. Tropsha, P. Gramatica, V.K. Gombar, The importance of being earnest: validation is the absolute essential for successful application and interpretation of QSPR models, QSAR & Combinatorial Science, 22 (2003) 69-77.

[2] P. Gramatica, External evaluation of QSAR models, in addition to cross‐validation: verification of predictive capability on totally new chemicals, Molecular informatics, 33 (2014) 311-314.

[3] A. Golbraikh, A. Tropsha, Beware of q2!, Journal of molecular graphics and modelling, 20 (2002) 269-276.
